# Identification of recombination hotspots and quantitative trait loci for recombination rate in layer chickens

**DOI:** 10.1186/s40104-019-0332-y

**Published:** 2019-02-26

**Authors:** Ziqing Weng, Anna Wolc, Hailin Su, Rohan L. Fernando, Jack C. M. Dekkers, Jesus Arango, Petek Settar, Janet E. Fulton, Neil P. O’Sullivan, Dorian J. Garrick

**Affiliations:** 1 Department of Animal Science, Iowa State University, Ames, IA 50010 USA; 2 Hy-Line International, Dallas Center, IA 50063 USA; 3 AL Rae Centre for Genetics and Breeding, Massey University, Palmerston North, 4442 New Zealand

**Keywords:** Layer chicken, QTL, Recombination, SNP

## Abstract

**Background:**

The frequency of recombination events varies across the genome and between individuals, which may be related to some genomic features. The objective of this study was to assess the frequency of recombination events and to identify QTL (quantitative trait loci) for recombination rate in two purebred layer chicken lines.

**Methods:**

A total of 1200 white-egg layers (WL) were genotyped with 580 K SNPs and 5108 brown-egg layers (BL) were genotyped with 42 K SNPs (single nucleotide polymorphisms). Recombination events were identified within half-sib families and both the number of recombination events and the recombination rate was calculated within each 0.5 Mb window of the genome. The 10% of windows with the highest recombination rate on each chromosome were considered to be recombination hotspots. A BayesB model was used separately for each line to identify genomic regions associated with the genome-wide number of recombination event per meiosis. Regions that explained more than 0.8% of genetic variance of recombination rate were considered to harbor QTL.

**Results:**

Heritability of recombination rate was estimated at 0.17 in WL and 0.16 in BL. On average, 11.3 and 23.2 recombination events were detected per individual across the genome in 1301 and 9292 meioses in the WL and BL, respectively. The estimated recombination rates differed significantly between the lines, which could be due to differences in inbreeding levels, and haplotype structures. Dams had about 5% to 20% higher recombination rates per meiosis than sires in both lines. Recombination rate per 0.5 Mb window had a strong negative correlation with chromosome size and a strong positive correlation with GC content and with CpG island density across the genome in both lines. Different QTL for recombination rate were identified in the two lines. There were 190 and 199 non-overlapping recombination hotspots detected in WL and BL respectively, 28 of which were common to both lines.

**Conclusions:**

Differences in the recombination rates, hotspot locations, and QTL regions associated with genome-wide recombination were observed between lines, indicating the breed-specific feature of detected recombination events and the control of recombination events is a complex polygenic trait.

**Electronic supplementary material:**

The online version of this article (10.1186/s40104-019-0332-y) contains supplementary material, which is available to authorized users.

## Background

Meiotic recombination occurs between homologous chromosomes and produces crossovers and gene conversions [1]. Characterizing patterns and rates of recombination is important for understanding genome-wide genetic diversity. Characterizing recombination frequency may have an impact on the interpretation of trait association studies (narrowing down the quantitative trait loci, also known as QTL regions) and on the consistency of marker effects estimates for genomic prediction. An understanding of the creation and loss of haplotypes caused by recombination during meiosis will enhance our ability to define optimal lengths of haplotypes and to reconstruct haplotype blocks, in order to improve imputation accuracy and genomic prediction accuracy. Recombination events are not evenly distributed across the genome, and their locations are strongly controlled by both *cis* and *trans* acting genes [2]. Recombination events occur more frequently in hotspots, which are defined as short intervals with significantly greater recombination rates compared to surrounding regions [3].

Recombination rates have been reported to differ by sex [4–6], chromosome [4, 7], species [8], and breed [9–11]. In humans the female recombination map is 1.7 times longer than the male recombination map [4, 12]. In the chicken the total length of male and female recombination maps are very similar, however recombination rates can be two-fold higher for microchromosomes than macrochromosomes [10, 13]. Furthermore, recombination rate is negatively correlated with the size of the chicken chromosomes [14]. Also, recombination rates have been found to be lower close to the centromere [8, 15], and positively correlated with GC content [10, 16]. The quality of map assembly [9] and family structure [17] can also impact the identification of recombination events.

Genome-wide association studies (GWAS) have identified several QTL that regulate genome-wide recombination rates in humans [6, 18, 19], mice [20], cattle [9, 11, 21], and plants [22]. These QTL include genetic variants in *RNF212* (ring finger protein 212), which controls genome-wide recombination rate in males and females [21, 23, 24] and *PRDM9* (PR domain containing 9), which has been identified as a regulator of recombination hotspots [21, 25]. It is reported that birds lack *PRDM9*, and their recombination is concentrated at gene promoters [26, 27]. The locations of recombination hotspots are conservative in birds, due to the lack of ortholog of *PRDM9* [28]*.*

Recombination rates assessed from high density SNP panels have been less thoroughly investigated in chickens. Crossover events were concentrated in promoters and CpG islands based on the whole-genome re-sequencing in 11 collared flycatcher [26]. Groenen et al. [10] identified genome sequence features that were correlated with recombination rate by genotyping ~ 10 K SNPs in three chicken populations. The objectives of the study herein were to determine the effect of number of SNP on detection of recombination, identify genome-wide recombination hotspots across the genome, identify genomic features (GC content and CpG islands) and QTL that influence recombination rates in two different purebred layer chicken breeds: white layers and brown layers.

## Methods

### Genotypes

Two chicken breeds were examined: white egg layers (WL) and brown egg layers (BL). The genotyping data was available for 448 half-sib families (282 sires and 166 dams) averaging 3.5 ± 2.7 birds per family for a total of 1200 birds in the WL line. This consisted of 969 sire-offspring pairs and 332 dam-offspring pairs. In BL line genotypes were available for 1717 half-sib families (621 sires and 1096 dams) with an average of 6.0 ± 4.9 birds per family for a total of 5108 birds. This consisted of 4719 sire-offspring pairs and 4573 dam-offspring pairs, respectively.

The WL birds were genotyped using the Affymetrix 580 K SNP chip. After removing SNPs with call rate < 0.95, minor allele frequency (MAF) < 0.025, or Mendelian inconsistency rate between parent-offspring > 0.05, a total of 172623 (173 K) segregating SNPs across 28 *Gallus gallus* (Galgal4.0 map assembly) autosomes (GGA) and the Z chromosome remained. This 173 K (173 K-WL) data was subsequently randomly trimmed down to 23 K (23 K-WL) to simulate a lower density panel. Sex chromosome information was not used for recombination detection but was included in GWAS analysis.

The BL birds were genotyped using an Illumina 42 K chip. Using the same quality criteria as for 173 K-WL, 22956 segregating SNPs were retained resulting in a 23 K-BL panel, including 5510 SNPs overlapping between the 173 K-WL and 23 K-BL panels.

Missing genotypes (~ 0.006% in WL and ~ 0.01% in BL) were imputed using FImpute [29].

### Identification of recombination events

Recombination events were determined on autosomes within half-sib families within each line using LINKPHASE version 3.0 [30]. Only half-sib families with genotyped parents and at least two genotyped offspring were used in the analysis. LINKPHASE3 utilizes linkage, and pedigree information, and applies a diploid Hidden Markov model using the Baum-Welch algorithm to improve haplotype reconstruction [17, 31]. LINKPHASE3 can detect putative map errors (e.g. misplaced SNPs on the map) based on a map confidence score, which combines information from recombination rates, parental genotyping errors, and genotype discrepancies in offspring [17]. Markers with a map confidence score less than 0.9 were considered as map errors and removed from the map assembly. Recombination intervals defined by pairs of informative heterozygous markers were reported for each parent-offspring pair. Total recombination rate was estimated for each non-overlapping 0.5-Mb window across macrochromosomes (GGA1-GGA5), intermediate chromosomes (GGA6-GGA10), and microchromosomes (GGA11-GGA28) [7, 13, 14]. The average recombination rate (*c*_*w*_) within each window (window recombination rate) was computed within line as:

1 $$ {c}_w=\left({\sum}_{i=1}^n{x}_i/{r}_i\right)/T $$

where *n* is the total number of recombination events observed on the corresponding whole chromosome, *x*_*i*_ is the length of overlapped region (in Mb) between the 0.5-Mb window and recombination interval *i*, *r*_*i*_ is the length (in Mb) of the recombination interval, and *T* is the total number of parent-offspring pairs. Window recombination rates were estimated across all parents and for sires and dams separately.

For each chromosome, the 10% of 0.5-Mb windows with the highest recombination rates were considered to be recombination hotspots regions and windows with no recombination detected across all individuals were considered to be cold spots. The genome-wide recombination number (GRN) was calculated for each parent as the average number of recombination events over 28 autosomes per meiosis. A t-test was used for significance testing of GRN between white and brown layers. The genome-wide hotspot usage (GHU) was calculated for each parent as the proportion of recombination events for that parent that fell within hotspots regions across the genome.

### Examining factors that affect observed recombination events

#### Marker density

The effect of marker density was evaluated only in the WL, by comparing whole-genome recombination number and 0.5-Mb window recombination rates from the 173 K-WL and the simulated lower density 23 K-WL panels.

#### Genomic inbreeding coefficient

Genomic inbreeding coefficients were calculated for each individual based on the observed against the expected (under Hardy-Weinberg Equilibrium) number of homozygous genotypes using PLINK v1.07 [32]. To minimize the probability of identifying inflated inbreeding coefficients, SNP data were pruned based on pair-wise LD within line by removing SNPs with *r*^2^ > 0.5 within each non-overlapping 50-SNP window. The relationship between genomic inbreeding coefficient and GRN was assessed in both lines.

#### Haplotype structure

Haplotype blocks with a fixed length of 0.5 Mb were constructed using the galGal4.0 map assembly. The number of common haplotype alleles was computed, which is the number of haplotype alleles with at least 1% frequency in a 0.5-Mb window. The number of common haplotype alleles was determined for every 0.5-Mb window in each of the 2 lines.

#### Chromosome size and GC content

The relationships of recombination rate with chromosome size (Mb), GC content, and CpG island density, in each 0.5-Mb window, was assessed in both lines. The GC content and CpG island density on each chromosome was calculated for each 0.5-Mb window using the hgTables tool from the UCSC genome browser [33].

### Estimating repeatability and heritability

Since recombination was detected for each parent-offspring pair, parents with multiple offspring had repeated records, which allowed estimation of heritability and repeatability of GHU and GRN. This was undertaken separately for each line using a repeatability model in ASReml3.0 [34], represented by:

2 $$ \boldsymbol{y}=\boldsymbol{Xb}+\boldsymbol{Zu}+\boldsymbol{Zp}+\boldsymbol{e} $$

where ***y*** is the vector of repeated GHU or GRN observations for each parent, ***b*** represents the means for each gender of parents treated as fixed effects, ***u*** is the vector of random animal genetic effects with $\mathrm{Var}\left(\boldsymbol{u}\right)=\boldsymbol{A}{\sigma}_a^2,$ where ***A*** is the pedigree relationship matrix among parents and ${\sigma}_a^2$ is the additive genetic variance, ***p*** is the vector of permanent environmental effects with $\mathrm{Var}\left(\boldsymbol{p}\right)=\boldsymbol{I}{\sigma}_p^2$, where ${\sigma}_p^2$ is the permanent environmental variance, ***X*** and ***Z*** are design matrices, and ***e*** is the vector of residual effects, with $\mathrm{Var}\left(\boldsymbol{e}\right)=\boldsymbol{I}{\sigma}_e^2$, where ${\sigma}_e^2$ is the residual variance.

Marker-based heritabilities of GHU and GRN were estimated using the average GHU or GRN for each parent in a weighted BayesC model with *π* equal to 0 [35, 36] implemented in GENSEL software version 4.4 [37, 38]. The model equation was:

3 $$ \boldsymbol{y}=\boldsymbol{Xb}+\boldsymbol{M}\boldsymbol{\alpha } +\boldsymbol{e} $$

where ***y***, ***X***, and ***b*** are as for model [2], ***M*** is a *n* × *m* matrix of SNP genotype covariates (coded 0, 1, or 2), *n* is the number of parents, *m* is the number of SNPs, ***α*** is a vector of random allele substitution effects for each SNP, and ***e*** is a residual effect with heterogeneous variance according to the number of offspring. The weighting factor *E*_*n*_ [39, 40] was used to account for heterogeneity of residual variance due to differences in the number of GHU or GRN observations contributing to the average GHU or GRN. This was calculated as:

4 $$ {E}_n=\frac{1-{h}^2}{\frac{1+\left(n-1\right)t}{n}-{h}^2} $$

where *h*^2^ is the narrow sense heritability estimated from pedigree, *t* is the repeatability, and *n* is the number of observations contributing to the average GHU or GRN for that parent.

The prior assumption of BayesC with *π* equal to 0 is that every SNP effect in [3] follows a normal distribution as below,

5 $$ {\alpha}_j\ {\displaystyle \begin{array}{c}i.i.d.\\ {}\sim \end{array}}\ N\left(0,{\sigma}_{\alpha}^2\right) $$

where *α*_*j*_ is the marker effect for SNP *j*, and ${\sigma}_{\alpha}^2$ is a common variance. Priors of the genetic and residual variance components required for BayesC were obtained from the pedigree based ASReml analysis. Markov chain Monte Carlo (MCMC) sampling over 55000 iterations, with the first 5000 samples discarded as burn-in, was used to estimate variance components and heritability.

### Identification of QTL affecting GRN

A genome-wide association study (GWAS) of average GRN for each parent was performed for each line using a weighted BayesB method [41, 42] implemented in GENSEL software [37, 38]. The model equations were similar to models [3, 4], except BayesB assumed a fraction *π* of SNP to have zero effects, and the non-zero distribution of SNP effects followed an identical and independent univariate-t distribution with null means, an inverse chi-squared prior for the SNP variances with degrees of freedom *υ*_a_, and scale parameter ${S}_{\mathrm{a}}^2$ [37, 38]. Priors for the SNP and residual variances were parameterized in terms of scale factors that were obtained from the genetic and residual variances estimated from pedigree based ASReml analyses. The MCMC had 55000 iterations, with the first 5000 samples discarded as burn-in. The GWAS was conducted across the genome (28 autosomes and sex chromosomes), separately for each line. The assumed values of *π* were 0.999 in WL and 0.99 in BL so that the number of fitted markers per MCMC iteration was about the same for the two lines, accounting for the difference in SNP density between the two lines.

The expected proportion of genetic variance (GV%) explained by each non-overlapping 1-Mb region is approximately 0.04% under a polygenic model. Those 1-Mb regions that explained at least 0.8% (> 20 times the expected %) of the genetic variance and their flanking regions on both sides of these regions (±1 Mb) were considered as QTL regions [9, 37, 43]. Posterior distributions for the proportion of genetic variance explained by each 1-Mb region were calculated from the post burn-in samples obtained from iterations of the MCMC chain [37].

The window posterior probabilities of association (PPA) of each QTL region, which is the percentage of samples for that region that contained at least one non-zero effect SNP for the trait, was also calculated. The SNP with the highest SNP posterior probability of inclusion (SPPI) was selected as the lead SNP for each QTL region. The SPPI was defined as the proportion of MCMC samples in which that SNP had been sampled with non-zero effects. Each lead SNP was fitted separately from the other SNP in the QTL region in a separate BayesB GWAS analysis described above, to estimate the GV% explained by that SNP [43]. Single SNP GWAS analyses were also conducted by fitting the lead SNPs as fixed effects in an animal model (with random animal effects and pedigree-based relationship matrix) using ASReml v3.0 [34], in order to evaluate their significance [44].

## Results and discussion

### Line comparison

**Fig. 1:**
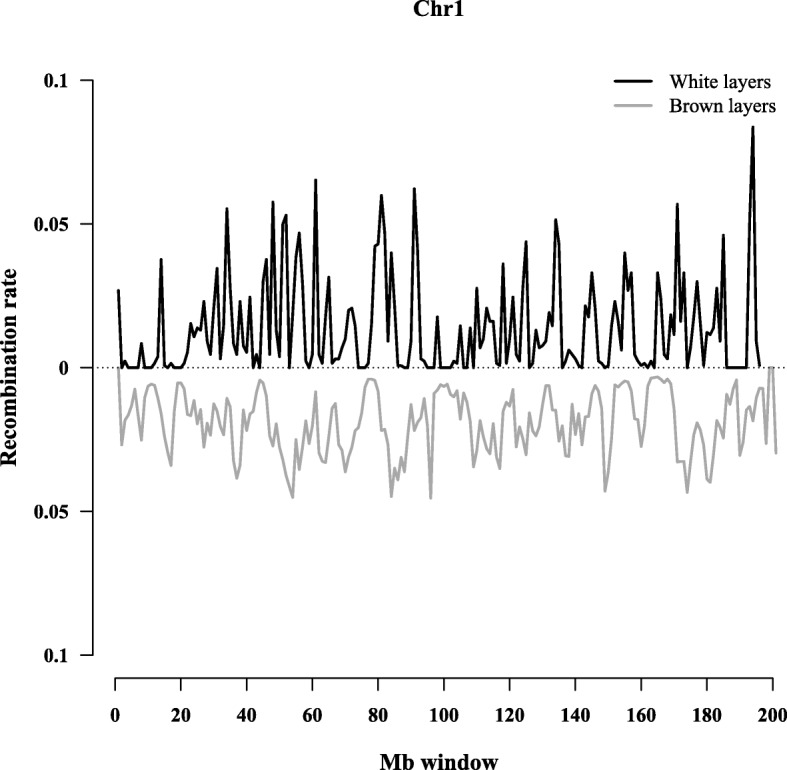
Recombination rate within 0.5-Mb windows estimated on GGA1. The black line corresponds to recombination rates estimated from 173 K SNPs in white layers. The grey line corresponds to recombination rate estimated from 23 K SNPs in brown layers

The average recombination rate across each 0.5-Mb window in each of the 28 chromosomes estimated from the 23 K-WL and 23 K-BL SNP data sets was calculated for the WL and the BL lines and is summarized in Table 1. The 0.5-Mb window recombination rates ranged from 0 to over 0.025 (average 0.0070 ± 0.010) in WL and from 0 to 0.047 (average 0.014 ± 0.012) in BL. The average window recombination rate in WL was about half that estimated in BL (*P* < 0.0001). Figure 1 uses GGA1 as an example to show 0.5-Mb window recombination rates in white and brown layers. The average window recombination rate on GGA1 was 0.013 ± 0.0070 in WL and in 0.014 ± 0.0096 BL. Window recombination rate varied along the chromosome and between lines. In some cases, windows with higher recombination rates for WL corresponded to a window with a lower recombination rate in BL and vice versa. Different window recombination rates and recombination landscapes were observed across the genome between the two lines (Additional file 1: Figure S1).

**Table 1:** Average recombination rate (SD) across all the 0.5-Mb windows in each chromosome of white (WL) and brown (BL) layers

GGA	Length,	WL				BL			
	Mb^a^	#SNP	Average	Male	Female	#SNP	Average	Male	Female
1	195.28	32,062	0.013 (0.0070)	0.012 (0.0057)	0.014 (0.0081)	4874	0.014 (0.0096)	0.013 (0.0088)	0.014 (0.0085)
2	148.81	20,372	0.0047 (0.0079)	0.0075 (0.0052)	0.0084 (0.0071)	3787	0.010 (0.0076)	0.012 (0.0074)	0.013 (0.0083)
3	110.45	18,370	0.0075 (0.010)	0.011 (0.0035)	0.016 (0.0072)	2725	0.011 (0.0096)	0.013 (0.0084)	0.014 (0.094)
4	90.22	14,496	0.0062 (0.0082)	0.0088 (0.0045)	0.012 (0.0073)	2279	0.011 (0.0091)	0.013 (0.0080)	0.013 (0.084)
5	59.58	10,448	0.0074 (0.0083)	0.010 (0.0056)	0.015 (0.0085)	1513	0.014 (0.0094)	0.016 (0.0087)	0.016 (0.088)
6	34.95	8548	0.0095 (0.0095)	0.014 (0.0061)	0.015 (0.0071)	864	0.013 (0.010)	0.015 (0.0086)	0.016 (0.0090)
7	36.25	6980	0.0013 (0.0038)	0.00035 (0.00056)	0.00069 (0.00086)	957	0.014 (0.009)	0.016 (0.010)	0.016 (0.010)
8	28.76	4678	0.0054 (0.0067)	0.0073 (0.0053)	0.0099 (0.0072)	681	0.016 (0.0015)	0.019 (0.018)	0.019 (0.016)
9	23.44	7237	0.0092 (0.0093)	0.012 (0.0058)	0.021 (0.0087)	605	0.018 (0.0014)	0.020 (0.013)	0.019 (0.015)
10	19.91	6248	0.012 (0.012)	0.016 (0.0067)	0.026 (0.014)	530	0.019 (0.014)	0.021 (0.011)	0.022 (0.013)
11	19.40	3845	0.0049 (0.0086)	0.0062 (0.0048)	0.0096 (0.0064)	507	0.015 (0.012)	0.016 (0.0097)	0.016 (0.0096)
12	19.90	4677	0.0053 (0.0078)	0.0057 (0.0069)	0.0062 (0.0069)	430	0.017 (0.012)	0.020 (0.011)	0.022 (0.013)
13	17.76	4062	0.0070 (0.0089)	0.0091 (0.0067)	0.014 (0.0089)	435	0.017 (0.011)	0.019 (0.011)	0.020 (0.011)
14	15.16	4043	0.010 (0.011)	0.013 (0.0091)	0.019 (0.013)	320	0.019 (0.017)	0.022 (0.015)	0.023 (0.018)
15	12.66	3265	0.0068 (0.011)	0.0098 (0.0072)	0.012 (0.0086)	298	0.020 (0.015)	0.024 (0.016)	0.024 (0.018)
16	0.54	21	0	0	0	10	0	0	0
17	10.45	1962	0.0082 (0.010)	0.094 (0.0090)	0.015 (0.015)	254	0.022 (0.015)	0.023 (0.016)	0.024 (0.017)
18	11.22	3244	0.012 (0.014)	0.016 (0.012)	0.019 (0.017)	261	0.023 (0.016)	0.026 (0.015)	0.025 (0.018)
19	9.98	2675	0.0018 (0.0030)	0.00068 (0.00078)	0.0057 (0.0065)	284	0.026 (0.015)	0.028 (0.015)	0.028 (0.017)
20	14.30	2162	0.0069 (0.0074)	0.0075 (0.0069)	0.010 (0.0078)	338	0.019 (0.013)	0.022 (0.013)	0.022 (0.014)
21	6.80	2439	0.016 (0.020)	0.023 (0.018)	0.023 (0.022)	206	0.034 (0.022)	0.037 (0.026)	0.035 (0.024)
22	4.08	929	0.00060 (0.0011)	0.00011 (0.00025)	0.00033 (0.0010)	73	0.023 (0.019)	0.024 (0.031)	0.025 (0.021)
23	5.72	1828	0.019 (0.020)	0.023 (0.022)	0.033 (0.033)	123	0.039 (0.021)	0.044 (0.022)	0.045 (0.029)
24	6.32	2320	0.015 (0.014)	0.018 (0.014)	0.022 (0.017)	180	0.037 (0.022)	0.043 (0.028)	0.041 (0.025)
25	2.19	556	0.0052 (0.011)	0.0031 (0.0063)	0.0066 (0.013)	36	0.036 (0.030)	0.044 (0.031)	0.043 (0.032)
26	5.33	1941	0.015 (0.016)	0.016 (0.012)	0.027 (0.025)	132	0.047 (0.027)	0.050 (0.031)	0.050 (0.031)
27	5.21	1918	0.025 (0.031)	0.032 (0.034)	0.024 (0.031)	111	0.023 (0.019)	0.028 (0.037)	0.029 (0.031)
28	4.74	1297	0.0035 (0.0073)	0.0020 (0.0037)	0.0049 (0.0085)	143	0.032 (0.032)	0.041 (0.036)	0.036 (0.034)

^a^Chromosome sizes based on galGal4 from UCSC website (https://genome.ucsc.edu)

Among the 190 and 199 recombination hotspots regions detected within 173 K-WL and 23 K-BL SNP sets respectively, 28 were common to both lines. There were 551 and 45 recombination cold spots detected in WL and BL, respectively, of which 22 were common to both lines. Overall, 14746 and 215808 recombination events were detected across the genomes in 1301 and 9292 meioses in WL and BL, respectively. The average GRN per meiosis were 11.33 in WL and 23.22 in BL. Several strategies were used to further investigate the cause of the higher estimate of recombination rate in the BL compared to the WL.

#### Marker density

GGA1 was used as an example (Additional file 2: Figure S2) to examine the impact of marker density on identification of recombination events. In WL the average 0.5-Mb window recombination rate on GGA1 using 23 K SNPs (randomly sampled from 173 K) was 0.011 ± 0.0056, which was significantly lower (*P* = 0.0046) than the average recombination rate obtained when using 173 K SNPs (0.013 ± 0.0070). The correlation of recombination rates on GGA1 based on these two different sized sets of segregating SNPs in white layers was 0.68. The average number of SNPs within a 0.5-Mb window was 103.3, 12.5, and 12.2 on GGA1 in 173 K-WL, 23 K-WL, and 23 K-BL, respectively. The average GRN detected in 23 K-WL was 8.58 ± 0.045, which was significantly lower (*P* < 0.0001) than the average GRN using 173 K-WL (11.89 ± 4.13), and both of which were lower than that detected in 23 K-BL (23.78 ± 4.37). A similar result was seen for other chromosomes, even if their size was different, and for the sex chromosome.

These results confirm the influence of marker density on the identification of recombination events, with more recombination events identified when individuals were genotyped with a panel of higher density. Denser markers provide more information along the genome, which would aid in locating recombination events within more precise intervals, and uncover recombination events which may have been undetectable with sparser markers.

#### Inbreeding coefficients

White layers had higher inbreeding coefficients (on average 0.082) than brown layers (0.031). The correlation between individual GRN and the genomic inbreeding coefficient was − 0.19 in WL and − 0.47 in BL. The regression coefficient between individual GRN and the genomic inbreeding coefficient was − 13.44 in WL and − 26.19 in BL. Crossover events that occur within a long chromosome segment of homozygous SNPs cannot be identified. Individuals with higher genomic inbreeding coefficients had more and longer lengths of homozygous regions, which might hinder the identification of recombination events. The 42 K panel had 23 K (55%) segregating SNPs in the BL, whereas the 580 K panel had only 173 K (30%) segregating SNPs in the WL. Although, panel creation is subject to ascertainment bias, neither panel was specifically created for either of these lines, and the segregation percentages imply that the WL was more inbred than the BL, in agreement with unpublished knowledge of the history of the line.

#### Haplotype structure

**Fig. 2:**
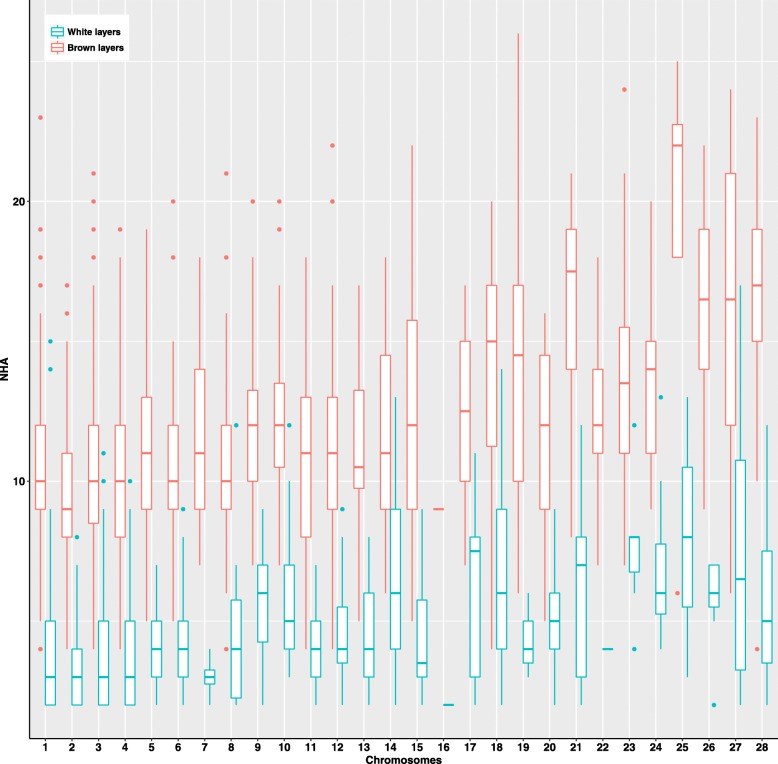
Distribution of the number of common haplotype alleles within 0.5-Mb window in white and brown layers

Figure 2 shows the distribution of the number of common haplotype alleles within 0.5-Mb windows across the 28 autosomes in both lines. The number of common haplotype alleles varied between chromosomes. The average number of common haplotype alleles in WL was 4.31 ± 2.25, which was significantly lower (*P* < 0.0001) than that in BL (10.90 ± 3.33), which is consistent with the WL being more inbred than the BL. The correlation between number of common haplotype alleles and window recombination rate was 0.49 in WL and 0.71 in BL. Recombination hotspots regions had significantly higher number of detected common haplotype alleles, compared to cold spots, in both lines. In WL, the numbers of common haplotype alleles were 6.60 ± 3.29 and 2.95 ± 1.21, in hotspots and cold spots regions, respectively. In BL, the numbers of common haplotype alleles were 15.78 ± 3.19 and 4.98 ± 0.46, in hotspots and cold spots regions, respectively.

Recombination breaks down haplotype alleles inherited from parental generations and creates new haplotype alleles. Fewer haplotype alleles were observed and more homozygous haplotype alleles were preserved in windows with lower recombination rate. The BL had a much higher detected recombination rate and a corresponding larger number of common haplotype alleles within 0.5-Mb window than WL. The prevalence of homozygous haplotype alleles is expected to reduce the number of identifiable recombination events.

Based on the above results, the observed window recombination rates and GRN differed significantly between the 2 lines, due to the breed-specific characteristics of recombination events. Groenen et al. [10] reported significant heterogeneity in recombination rates between three different chicken populations. Weng et al. [9] reported differences in detected recombination between the Angus and Limousin breeds of beef cattle. It is possible that “true” recombination rates are more similar between lines than are apparent in our study because not all recombination events can be identified. That is, breed-specific characteristics of detected recombination events could be due to population structure (e.g. inbreeding levels) and genomic structure (e.g. haplotype structure). Moreover, genetic variants that regulate recombination in different breeds might be different. Different QTLs associated with genome-wide recombination were detected in the Angus and Limousin cattle breeds [9].

### Recombination between sexes

**Fig. 3:**
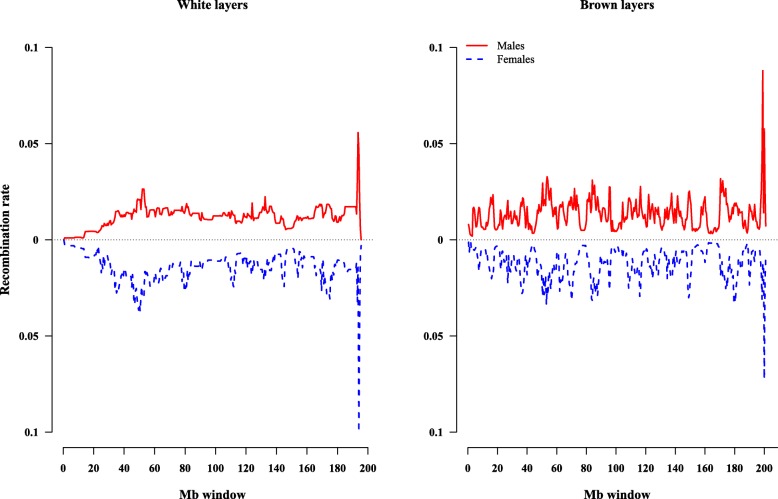
Recombination rate within 0.5-Mb window estimated on GGA1 for males (red solid line) and females (blue dashed line) in white and brown layers

**Fig. 4:**
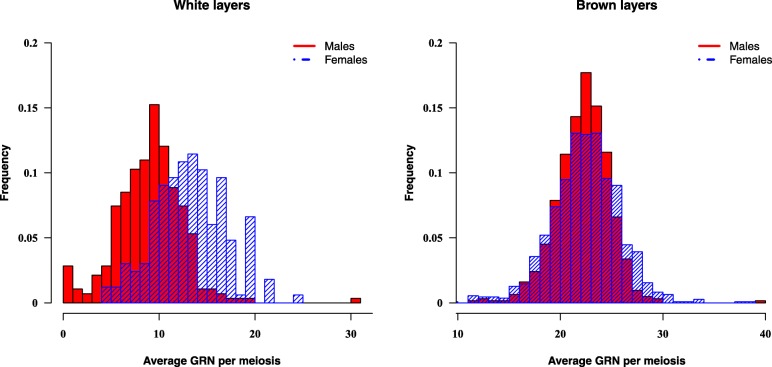
Distributions of the average GRN per meiosis in males (red) and females (blue) in white and brown layers

Table 1 presents 0.5-Mb window recombination rates separately for sire and dam families. The difference in recombination rates was significant between genders for both WL (sire = 0.0099 ± 0.0077; dam = 0.013 ± 0.010; *P* < 0.0001) and BL (sire = 0.016 ± 0.013; dam = 0.017 ± 0.013; *P* = 0.0076). In Fig. 3, the recombination patterns were different between sires and dams on GGA1 in both lines. Window recombination rates varied along the chromosome in both sires and dams, and most locations of recombination hot and cold spots regions were inconsistent for both sexes in WL (Additional file 3: Figure S3) and BL (Additional file 4: Figure S4). Figure 4 shows the average GRN per meiosis of sires and dams in WL and BL. The average GRN per meiotic event differed between sexes in both WL (*P* < 0.0001) and BL (*P* = 0.040).

In WL, the average size of half-sib families was 4.0 ± 3.0 for sires, and 2.5 ± 1.5 for dams. In BL, the average size of sire families was 8.0 ± 5.7 and 4.7 ± 4.0 for dam families. Random samples of 10 sire and 10 dam half-sib families (10 offspring per family) were used to recalculate 0.5-Mb window recombination rates and average GRN, separately in sires and dams, in order to avoid the impact of numbers of observations and family sizes on identification of recombination events between the sexes. This did not change our findings, as the average window recombination rate was still higher in dams than sires in both WL (*P* = 0.0001) and BL (*P* = 0.0052). Based on the random samples, dams (16.36 ± 1.10) had higher GRN than sires (11.11 ± 0.52) in WL. In BL, GRN was 23.27 ± 0.20 in dams, and 22.38 ± 0.16 in sires.

In conclusion, in this study, differences between sexes appear not to result from inbreeding coefficients or haplotype structures. The observed window recombination rates and the average GRN were significantly greater in dams than in sires in both lines. The difference in WL was larger than previously reported in layer chickens [10]. Groenen et al. [10] only observed 3% difference between sexes in the WU population. Different recombination rates between the sexes, i.e. females having higher recombination rates than males, have been identified in *Drosophila* [45], mice [5], cattle [11, 46], sheep [47, 48], plants [49], and humans [4, 6]. Higher recombination rate in males were observed in pseudoautosomal regions in mouse, however, the results might be influenced by the limited resolution of the pseudoautosomal markers used in the study [50]. Petkov et al. [5] reported that crossover interference is the main factor causing sex differences in recombination rate. The timing of meiosis differs significantly between males and females in mammals [51], which could contribute to the sex-specific manner of recombination. Male germ cells enter meiosis and continue actively dividing after puberty, while female germ cells enter meiosis and stop dividing within the fetal ovary [51]. Also, genetic variants that control recombination in females and males might be different. Kong et al. [52] used the Icelandic genealogy database to identify three sex-specific variants associated with male genome-wide recombination rate, and seven sex-specific variants associated with female recombination rate. However, Kadri et al. [46] found many shared variants between sexes in cattle and a high genetic correlation between male and female recombination rate.

### Chromosome size and GC content

The average window recombination rates changed depending on chromosome size, with recombination rates per 0.5-Mb on the microchromosomes being significantly higher than on the macrochromosomes for both white (*P* = 0.011) and brown layers (*P* < 0.0001). The average 0.5-Mb window recombination rates in WL were: macrochromosomes = 0.0067 ± 0.0092, intermediate chromosomes = 0.0069 ± 0.0091, and microchromosomes = 0.0085 ± 0.013. Whereas, in BL the respective window recombination rates were: 0.011 ± 0.0086, 0.016 ± 0.013, and 0.022 ± 0.018 on macrochromosomes, intermediate chromosomes and microchromosomes. A negative correlation between the average window recombination rate and chromosome length was observed in both the white (− 0.16) and brown (− 0.50) layer lines. Rodionov [53] and Groenen et al. [10] also observed higher recombination rates in microchromosomes compared to macrochromosomes. In this study, GGA1-GGA5 were considered as macrochromosomes, while GGA11-GGA28 were defined as microchromosomes [7, 13, 14]. Although Rodionov [53] and Groenen et al. [10] considered GGA1–8 as macrochromosomes, results still indicate that recombination rate is negatively correlated with chromosome size [14]. Kong et al. [4] and Weng et al. [9] found similar results in humans and beef cattle respectively.

In WL, the correlation of recombination rate was 0.13 with both GC content and CpG island density (Additional file 5: Figure S5). In BL, correlations between recombination rate with GC content and CpG island density were both 0.29, which is higher compared to WL. Recombination hotspots regions differed significantly from cold spots regions in both GC content (*P* = 0.046) and CpG island density (*P* = 0.042) in both lines. The association of recombination with GC content and CpG island density was stronger in BL than in WL. Although it is known that recombination rate is related to genome structure, the strength of this relationship varies among organisms. For example, GC content is positively correlated with recombination rate in mammals [8, 16] but shows weak or no correlations in plants [15, 22]. The 0.5-Mb windows with higher recombination rates had higher GC content. According to the “biased gene conversion” hypothesis [54], recombination hotspots become GC rich regions [14].

### Genome regions associated with GRN

GRN was repeatable, with repeatability estimates of 0.24 ± 0.020 and 0.21 ± 0.033 in white and brown layers, respectively. Heritability was 0.17 ± 0.022 in WL and 0.16 ± 0.0037 in BL. Marker-based heritability estimates of GRN were similar to the pedigree-based estimates, being 0.15 ± 0.0014 and 0.13 ± 0.0013 in WL and BL, respectively. Estimates of heritabilities in layer chickens were lower than those reported in beef cattle, dairy cattle, and humans. Weng et al. [9] reported a pedigree-based heritability of GRN of 0.26 in Angus and 0.23 in Limousin. Sandor et al. [21] reported a heritability estimate of 0.22 in Dutch Holstein-Friesian bulls. The heritability of recombination rate was reported to be 0.30 in humans [55].

The number of recombination events was treated as a quantitative trait to map genetic variants that influence recombination rates. The proportion of genetic variance explained by each 1-Mb region across the genome in WL and BL is presented in Additional file 6: Figure S6 and Additional file 7: Figure S7. Tables 2 and 3 show the proportion of genetic variance explained, PPA of QTL regions, MAF, physical position, significance of the SNPs with highest effect, and the list of nearby candidate genes. Since it has been shown that the location of the causal mutation could be extended to 1 Mb on either side of the informative 1 Mb QTL regions, neighboring regions were combined for analysis [9, 37, 43]. In general, 14 QTL on eight chromosomes for recombination rate were identified in WL. Only 6 QTL on four chromosomes were identified in BL. The GV% explained by significant QTL regions ranged from 1.0% to 8.4% in WL, and from 0.8% to 19.7% in BL. No common QTL regions were identified across the two lines.

**Table 2:** Description of 1-Mb windows that explained more than 0.8% of genetic variance and significant SNPs within those windows for genome-wide recombination number in white layers

GGA_Mb	# SNP	GV%^a^	WPPA^b^	Most significant SNP^c^	SPPI^d^	GV% SNP	MAF^e^	Position, Mb	*P-* value^f^	Candidate gene
1_54	126	1.00	0.27	AX-80768024	0.02	0.05	0.132	54.56	0.003	*TDG, NFYB*
1_84	112	8.39	0.54	AX-80984549	0.52	8.38	0.151	84.06	0.04	*RPL24, IMPG2*
1_158	165	1.00	0.23	AX-75313537	0.01	0.42	0.349	158.73	0.005	
1_162–165	801	4.39	0.89	AX-75325622	0.02	1.3	0.189	165.07	0.006	*RGCC, WBP4, LECT1*
1_171	198	1.00	0.34	AX-75341236	0.02	0.1	0.316	171.68	0.03	*CCNA1*
2_76–77	408	4.13	0.76	AX-76154814	0.02	0.15	0.346	77.43	0.06	*ANKH*
2_81–82	196	3.91	0.64	AX-76163810	0.01	0.38	0.312	82.25	0.04	
3_85	468	3.92	0.79	AX-76572482	0.01	0.16	0.357	86.15	0.04	*PRIM2*
4_44	183	7.93	0.56	AX-76669056	0.48	7.88	0.126	44.57	0.08	*EPGN, AREGB, EREG*
5_13	398	2.23	0.64	AX-76779213	0.01	0.26	0.355	13.59	0.02	*IGF2, MRPL23, RAG1, RAG2*
10_1	382	1.08	0.39	AX-75574022	0.01	0.1	0.472	1.06	0.03	*RPS17L*
13_3	166	2.37	0.50	AX-75754286	0.01	0.77	0.465	3.62	0.02	
20_5	283	1.02	0.35	AX-76217814	0.01	0.2	0.316	5.09	0.02	*YWHAB*
20_10	189	1.15	0.29	AX-76199322	0.03	0.12	0.385	10.96	0.005	*SPO11*

^a^GV%, proportion of the genetic variance explained by window or SNP

^b^WPPA, window posterior probability of association

^c^Most significant SNP, SNP with the highest SPPI in the 1 Mb region

^d^SPPI, SNP posterior probability of inclusion

^e^MAF, minor allele frequency

^f^*P-*value, the significance of the single SNP as a fixed effect in ASREML

**Table 3:** Description of 1-Mb windows that explained more than 0.8% of genetic variance and significant SNPs within those windows for genome-wide recombination number in brown layers

GGA_Mb	# SNP	GV%^a^	WPPA^b^	Most significant SNP^c^	SPPI^d^	GV% SNP	MAF^e^	Position, Mb	*P*-value^f^	Candidate gene
1_15	22	0.83	0.49	Gga_rs13713650	0.34	0.10	0.38	15.28	0.022	*CBLL1*
1_66	19	5	0.57	Gga_rs13879050	0.45	0.10	0.39	66.67	0.06	*RECQL*
2_73	16	19.74	0.80	Gga_rs14203992	0.74	0.20	0.35	73.74	0.07	*CDH10*
2_138,140–141	72	12.16	0.29	Gga_rs13794969	0.17	2.24	0.39	138.20	0.005	*MYC*
4_85	33	2.21	0.80	Gga_rs14498387	0.67	0.54	0.29	85.48	0.07	*RNF212, MAEA, FGFRL1, MRPL35*
12_2	23	0.83	0.61	Gga_rs14032471	0.50	0.46	0.071	2.72	0.1	*FANCD2, GNAI2*

^a^GV%, proportion of the genetic variance explained by window or SNP

^b^WPPA, window posterior probability of association

^c^Most significant SNP, SNP with the highest SPPI in the 1 Mb region

^d^SPPI, SNP posterior probability of inclusion

^e^MAF, minor allele frequency

^f^*P*-value, the significance of the single SNP as a fixed effect in ASREML

A total of 20 positional candidate genes were located within and/or near the QTL regions in WL, while 10 candidate genes were found in BL. No candidate genes were identified near QTL on GGA1 at 158 Mb, GGA2 at 81–82 Mb, or GGA13 at 3 Mb in WL.

#### Candidate genes identified in WL

One of the strongest candidate genes, *SPO11* (SPO11 meiotic protein covalently bound to DSB homolog), was identified on GGA20 near 10 Mb in WL. *SPO11* produces a meiosis-specific protein, which could initiate recombination [2]. Guillon et al. [56] reported that *SPO11* induces double-strand break during meiosis in mice. Weng et al. [9] identified *SPO11* as a positional candidate gene for genome-wide recombination number in Limousin beef cattle.

One candidate gene was identified on GGA3, namely *PRIM2* (primase, DNA, polypeptide 2), which is located at 86.05–86.13 Mb. It was reported that *PRIM2* is active in both the initiation of DNA replication and synthesis [57]. The positional candidate genes on chromosome 5 at 13 Mb were *IGF2* (insulin-like growth factor 2)*, MRPL23* (mitochondrial ribosomal protein L23)*, RAG1* (recombination activating gene 1), and *RAG2* (recombination activating gene 2). *IGF2* is a growth factor that controls hormone activity, regulation of mitosis, and cell differentiation. *MRPL23* is involved in the cellular process of translation and integration of membrane and ribosome. The proteins *RAG1* and *RAG2* carry out the formation of double-stranded breaks at recombination signal sequences [58].

#### Candidate genes identified in BL

The signal near *RNF212* on GGA4 was identified as influencing GRN in BL. The sequence variants in *RNF212* are reported to affect genome-wide recombination rate in males and females in humans [18, 23]. Its significant impact on recombination rate has also been detected in cattle [9, 21, 46].

One candidate gene, *RECQL* (RecQ protein-like) located at 65.47–65.49 Mb, was found on GGA1. *RECQL* is involved in various types of DNA repair, including mismatch repair, nucleotide excision repair, and direct repair [59]. The window containing *RECQL* was associated with GRN in Angus cattle [9]. GGA2 had two candidate genes: *CDH10* (cadherin 10, type 2) located at 72.18–72.27 Mb, and *MYC* (v-myc avian myelocytomatosis viral oncogene homolog) located at 139.31–139.32, which plays a critical role in DNA replication, cell growth and cell cycle progression. The candidate gene of *FANCD2* (Fanconi anemia, complementation group D2) was identified on chromosome 12 at 2 Mb, which promotes gene conversion and DNA repair.

Only two genes previously identified as influencing recombination rate were present in regions detected in this study: *SPO11* in WL and *RNF212* in BL. Other promising candidate genes include *RAG1*, *RAG2*, and *RECQL*, although further investigation is required. The different mapping results between WL and BL, and between chickens and other organisms (e.g. cattle, humans, mice and plants) suggest that recombination is a species-specific polygenic trait.

### Genome-wide hotspot usage

Individual GHU ranged from 0 to 50% in WL and 0–83.8% in BL. The average GHU in WL was 9.6% ± 5.9%, which was significantly different (*P* < 0.0001) from BL (20.3% ± 5.4%). Gender effects on GHU were significant in both lines (WL: *P* = 0.021; BL: *P* = 0.042). The average GHU was 9.1% ± 6.2% for sires and 10.4% ± 5.2% for dams in WL. The average GHU was 20.2% ± 4.6% for sires and 20.9% ± 5.8% for dams in BL. Heritability and repeatability estimates were 0.10 ± 0.041 and 0.14 ± 0.037 in WL and 0.10 ± 0.015 and 0.14 ± 0.014 in BL. These two estimated heritabilities were lower than the 0.21 value computed in cattle [21]. The GHU was heritable in human with a estimated narrow sense heritability of 0.23 [60].

Several studies have shown that the *PRDM9* gene controls activation of mammalian recombination hotspots [21, 25]. Particularly, Sandor et al. [21] observed large peaks on chromosome X in cattle at the position of two *PRDM9* paralogues. In this study, GWAS on GHU identified 6 and 11 QTL regions (each explaining > 0.5% genetic variance) in WL and BL (results not reported). In WL, 6 QTL regions were identified on GGA3, 9, 10, 20, 21, and 28. In BL, 11 significant QTL regions were located on the Z chromosome. No QTL regions overlapped between the two lines, and no candidate genes were identified due to limited gene annotation information. Genetic variants controlling GHU might differ between breeds and species.

## Conclusion

Genome-wide recombination patterns and rates were characterized in two egg laying chicken breeds: white layers and brown layers. A large number of recombination events and recombination hotspots regions were identified. The BL were genotyped at lower density but had a higher number of detected recombination events than the WL, which were genotyped at higher density. Marker density can influence the identification of recombination events but different detectable recombination events were found between the 2 lines even with comparable SNP densities. Differences in the recombination rates (numbers) and hotspot locations were observed between lines, showing the breed-specific feature of detected recombination events. This breed-specific characteristic of detected recombination events may be due to inbreeding levels, haplotype structures, genetic variants, or other differences between the two breeds studied. The frequencies of recombination events were found to differ by sex. Dams had higher window recombination rates and more recombination events per meiosis than sires. Window recombination rate showed a negative correlation with chromosome size and positive correlations with GC content, and CpG island density in the two lines. Several QTL regions associated with genome-wide recombination were identified in the two breeds, suggesting that the control of recombination events is a complex polygenic trait.

## Additional files

Additional file 1: **Figure S1**. Variation in recombination rate within 0.5-Mb windows across the 28 autosomes (except GGA6). The black line corresponds to recombination rates estimated from segregating 173K SNPs in WL. The grey line corresponds to recombination rate estimated from segregating 23K SNPs in BL. (PPTX 127 kb)

Additional file 2: **Figure S2.** Recombination rate within 0.5-Mb estimated using segregating 173K SNPs (black line) and 23K SNPs (green line) in white layers on GGA1. (PDF 6 kb)

Additional file 3: **Figure S3.** Variation in recombination rate in males (red solid line) and females (blue dashed line) within 0.5-Mb windows across the 28 autosomes in WL (except GGA16). (PPTX 152 kb)

Additional file 4: **Figure S4.** Variation in recombination rate in males (red solid line) and females (blue dashed line) within 0.5-Mb windows across the 28 autosomes in BL (except GGA16). (PPTX 164 kb)

Additional file 5: **Figure S5.** Relationship of GC content and CpG island density with recombination rate within 0.5-Mb window across chromosomes in WL and BL. (PDF 54 kb)

Additional file 6: **Figure S6.** Proportion of genetic variance explained by 1-Mb regions across the genome for GRN in WL. (PDF 10 kb)

Additional file 7: **Figure S7.** Proportion of genetic variance explained by 1-Mb regions across the genome for GRN in BL. (PDF 10 kb)

## Abbreviations

BL: Brown-egg Layers
GGA: *Gallus Gallus* Autosomes
GHU: Genome-wide Hotspot Usage
GRN: Genome-wide Recombination Number
GV%: Proportion of Genetic Variance
GWAS: Genome-Wide Association Study
MCMC: Markov Chain Monte Carlo
PPA: Posterior Probabilities of Association
QTL: Quantitative Trait Loci
SNP: Single Nucleotide Polymorphisms
SPPI: SNP Posterior Probability of Inclusion
WL: White-egg Layers
