# Supplementary figures and images for: Identification of recombination hotspots and quantitative trait loci for recombination rate in layer chickens
Source: J Anim Sci Biotechnol. 2019 Feb 26;10:20. doi: 10.1186/s40104-019-0332-y (PMC6390344; doi:10.1186/s40104-019-0332-y)

## Slide 1
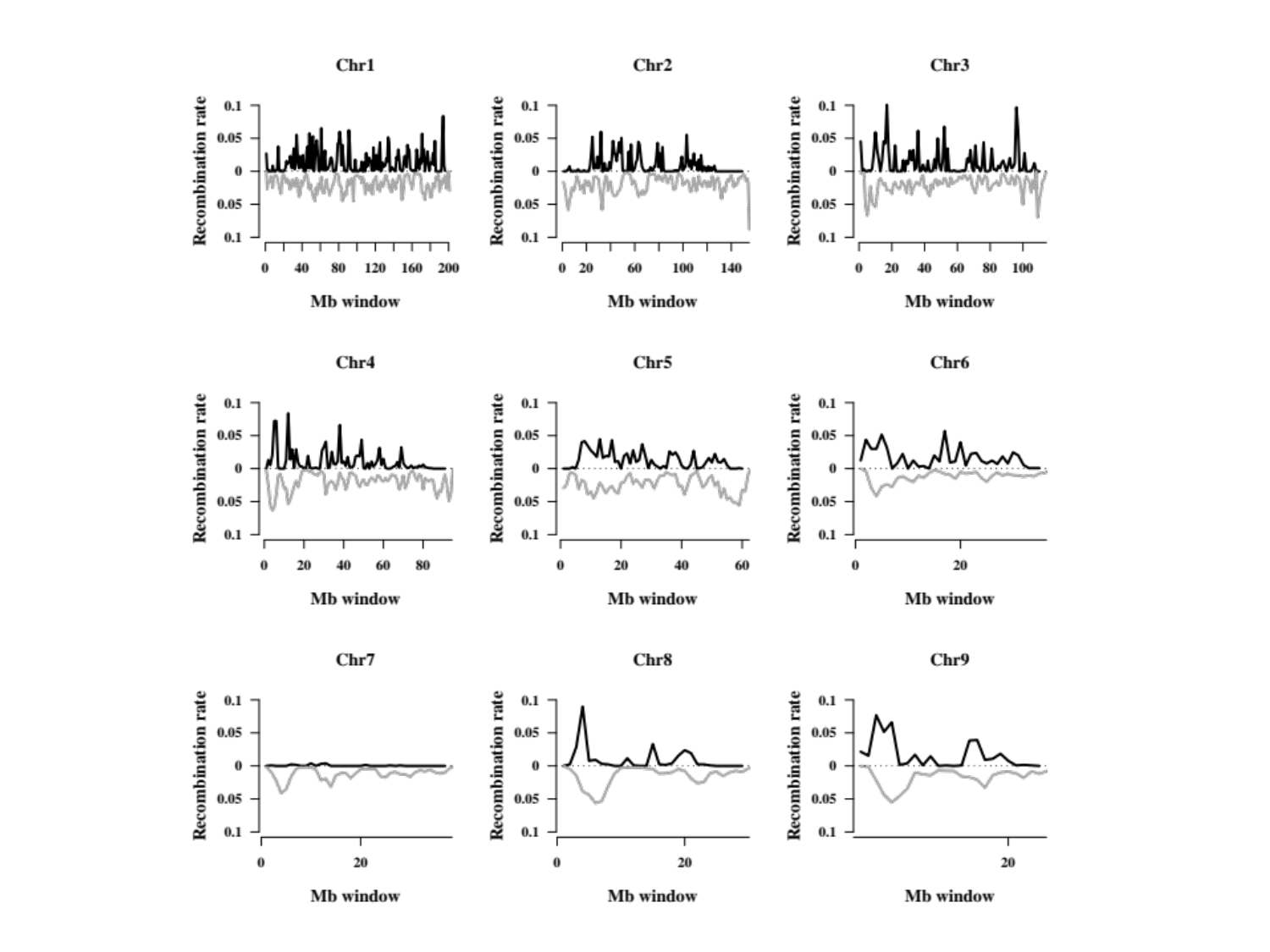

## Slide 2
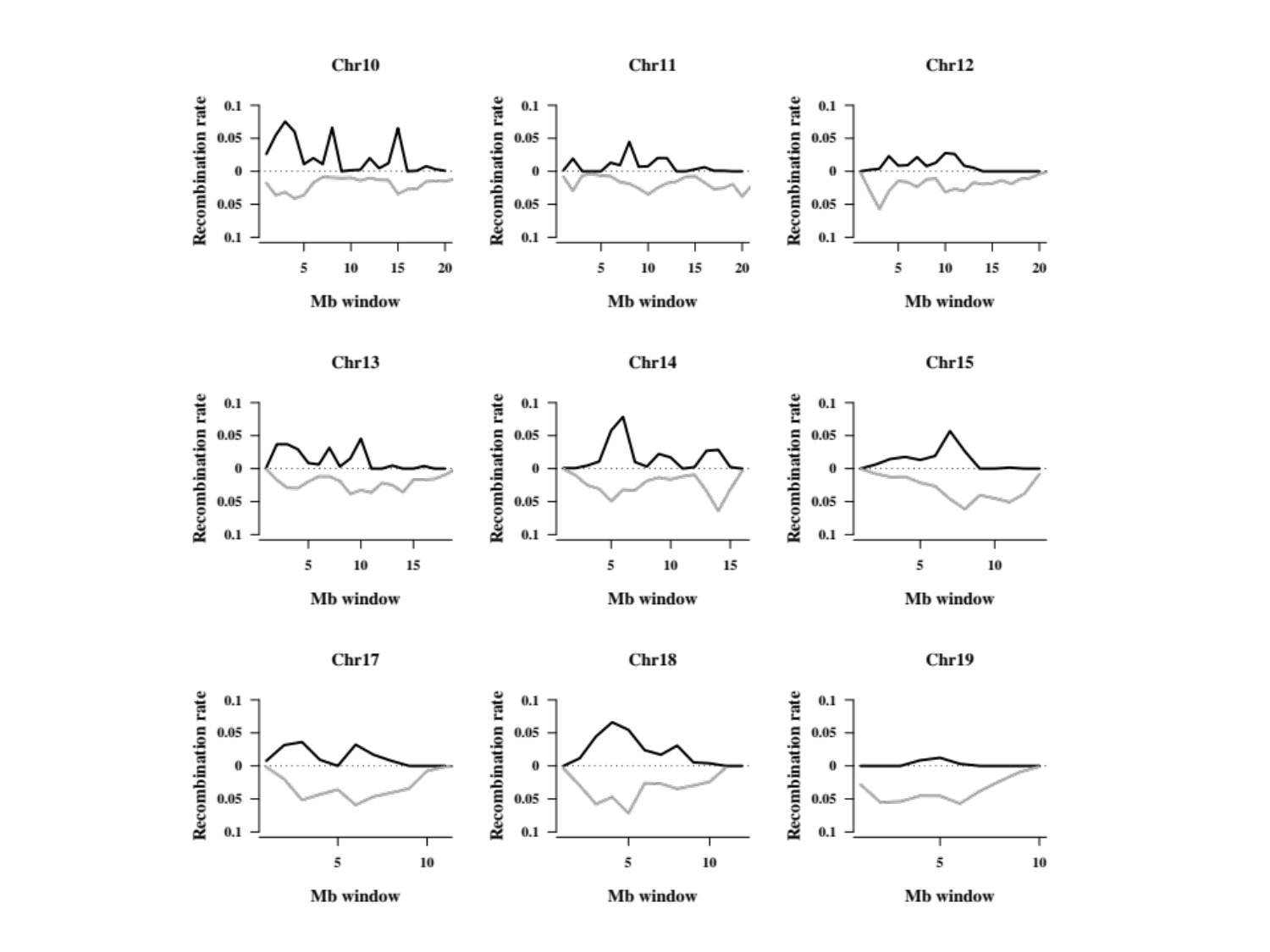

## Slide 3
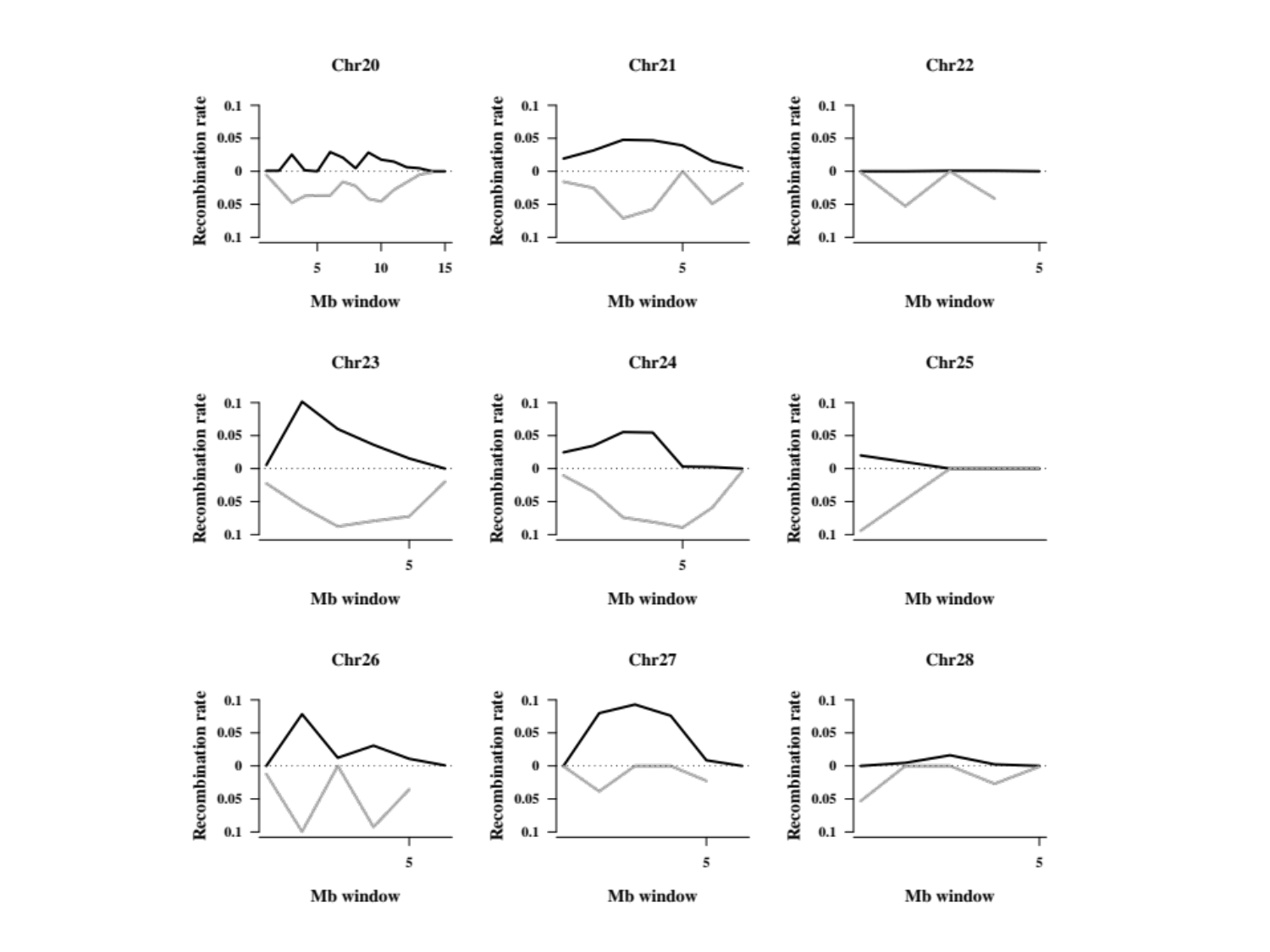

Supplement: Supplementary file 1 — Figure S1. Variation in recombination rate within 0.5-Mb windows across the 28 autosomes (except GGA6). The black line corresponds to recombination rates estimated from segregating 173K SNPs in WL. The grey line corresponds to recombination rate estimated from segregating 23K SNPs in BL. (PPTX 127 kb) [file 40104_2019_332_MOESM1_ESM.pptx]

Chr1

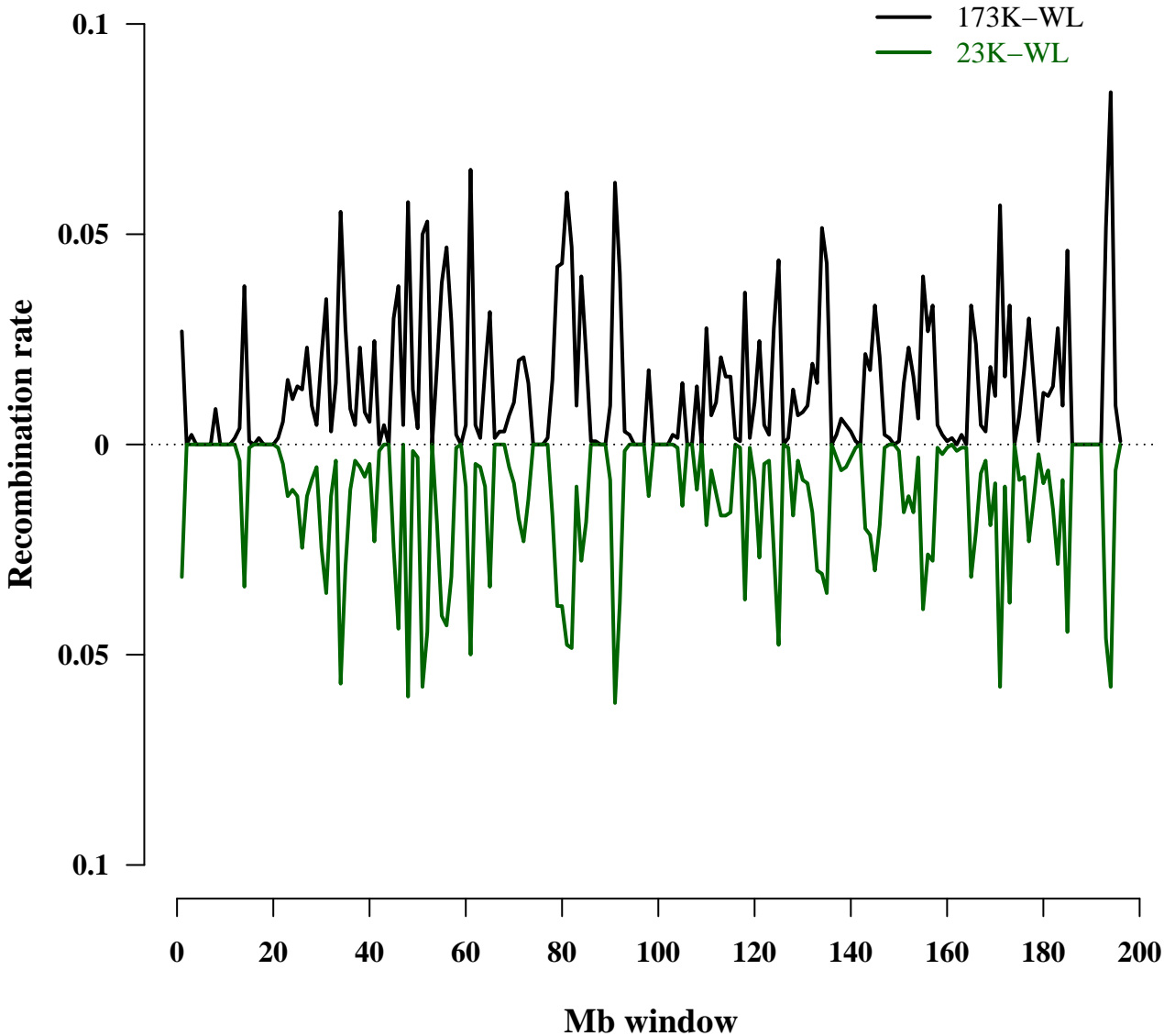

Supplement: Supplementary file 2 — Figure S2. Recombination rate within 0.5-Mb estimated using segregating 173K SNPs (black line) and 23K SNPs (green line) in white layers on GGA1. (PDF 6 kb) [file 40104_2019_332_MOESM2_ESM.pdf]

## Slide 1
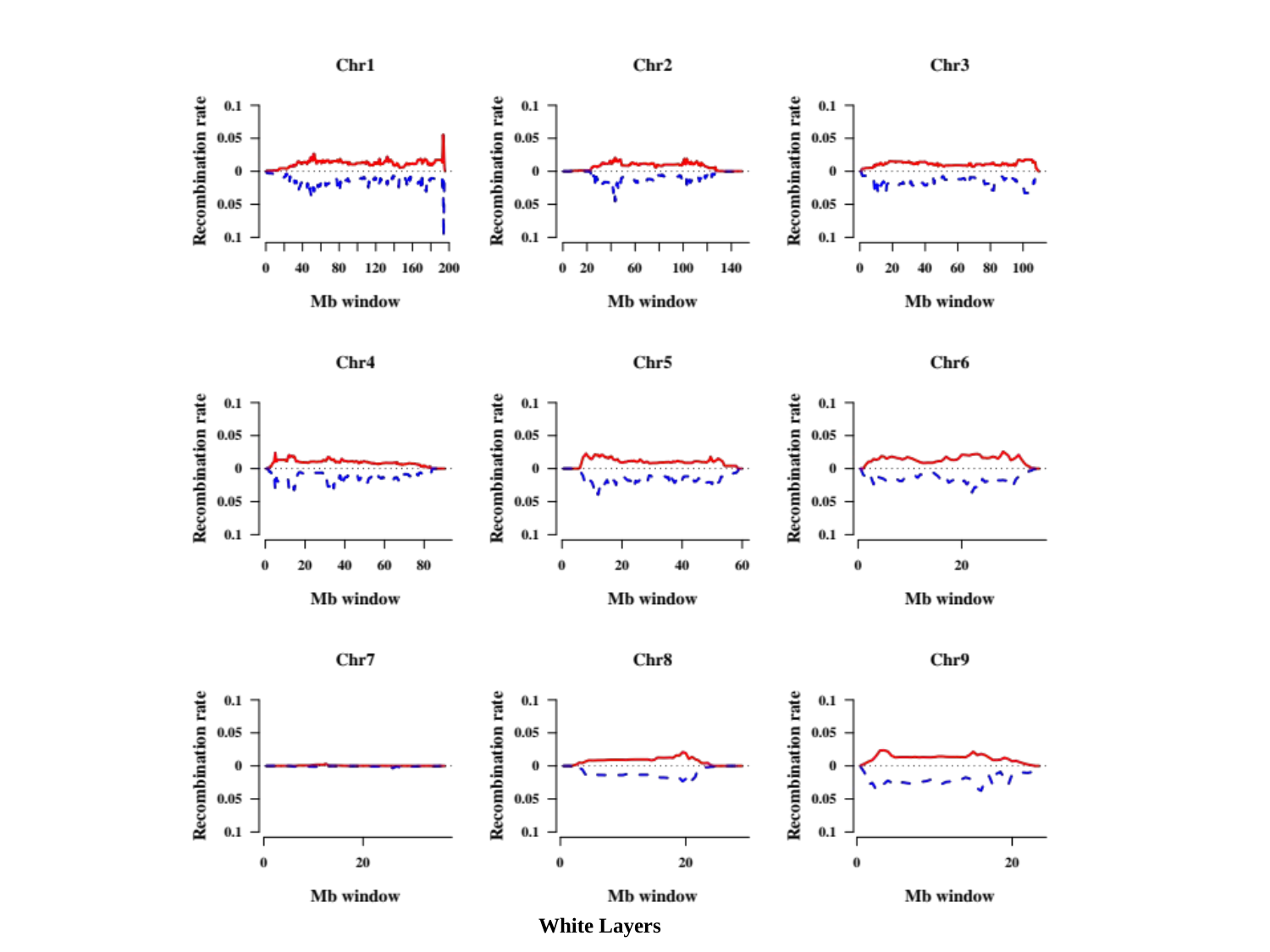

White Layers

## Slide 2
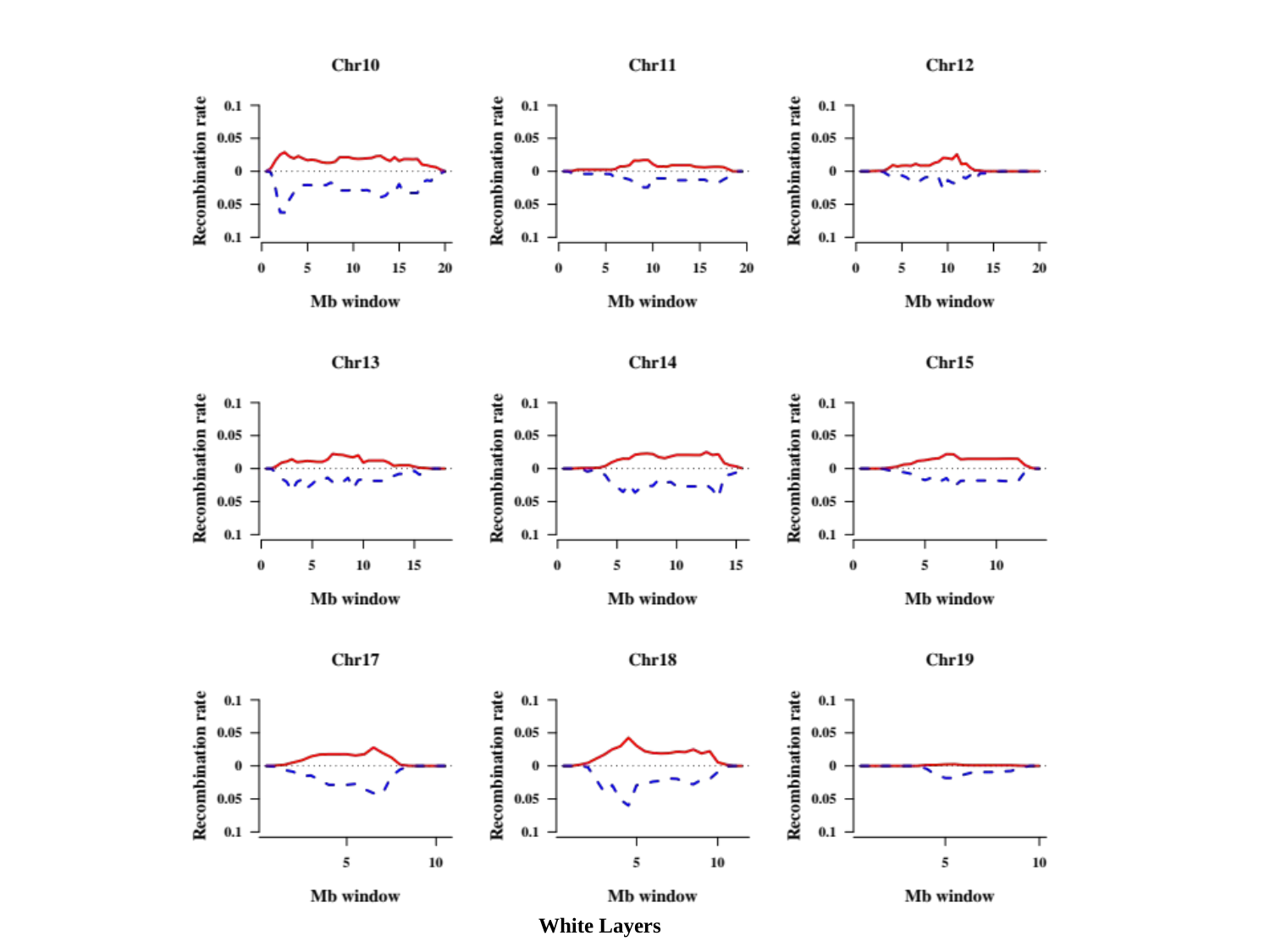

White Layers

## Slide 3
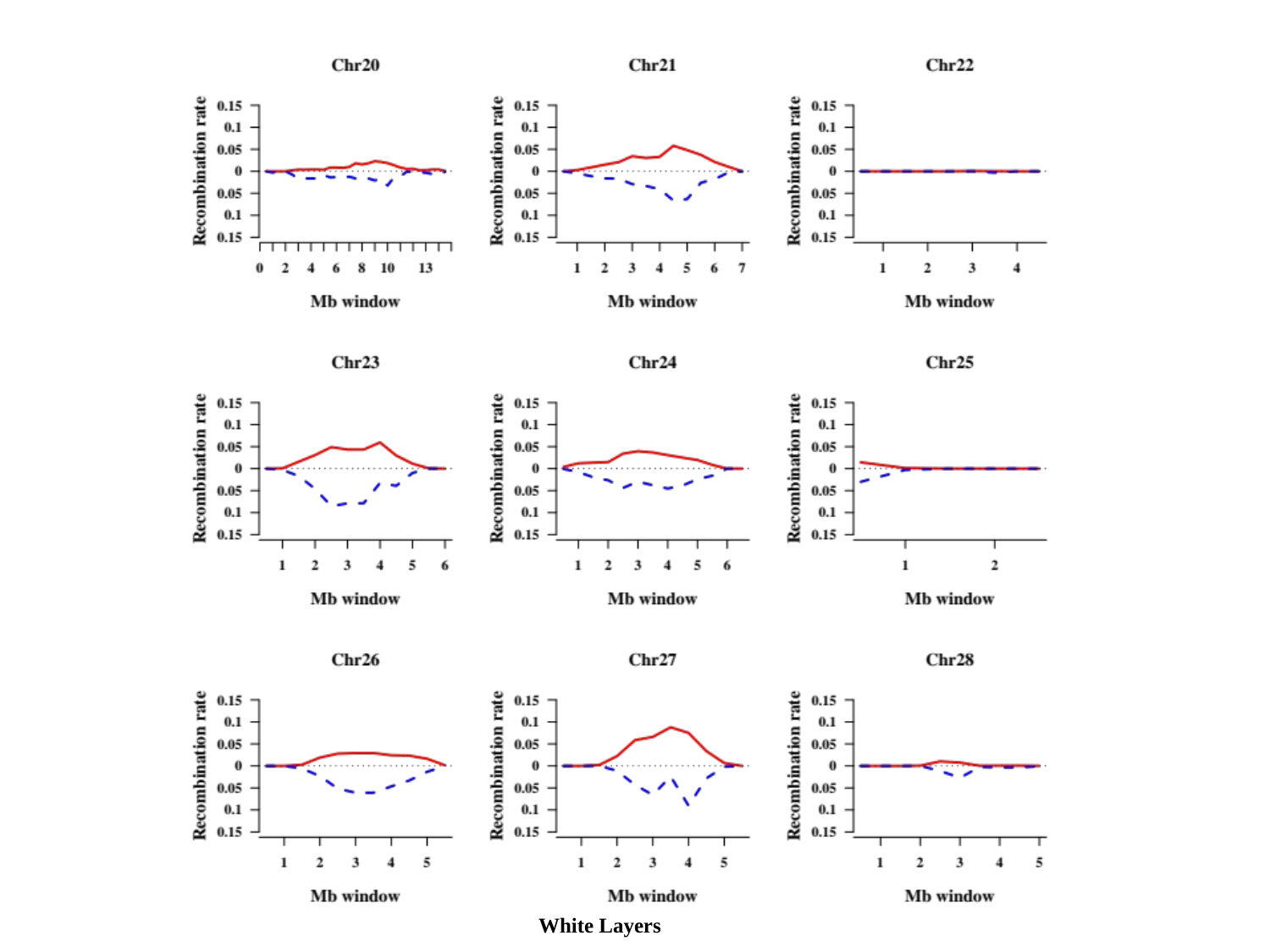

White Layers

Supplement: Supplementary file 3 — Figure S3. Variation in recombination rate in males (red solid line) and females (blue dashed line) within 0.5-Mb windows across the 28 autosomes in WL (except GGA16). (PPTX 152 kb) [file 40104_2019_332_MOESM3_ESM.pptx]

## Slide 1
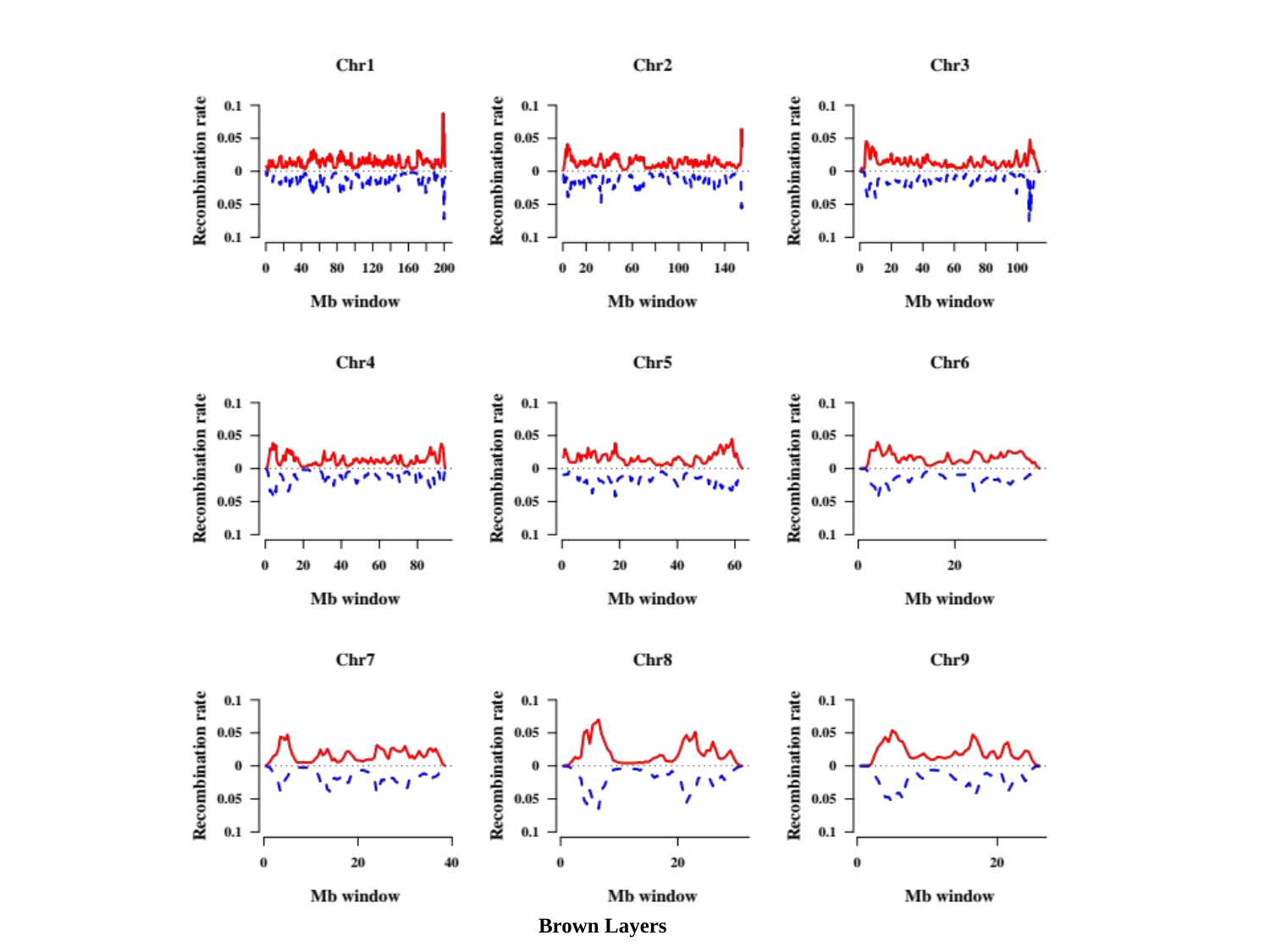

Brown Layers

## Slide 2
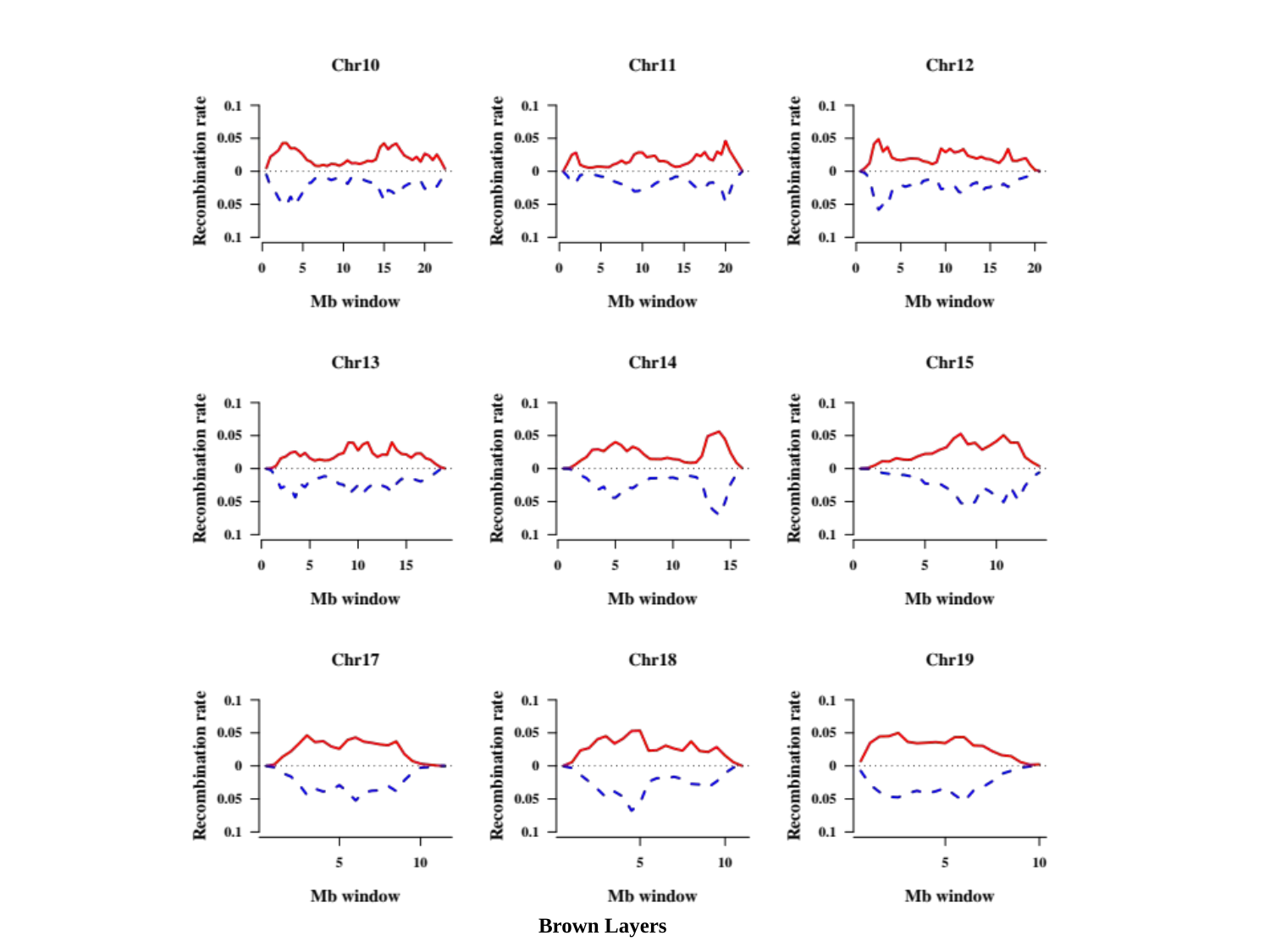

Brown Layers

## Slide 3
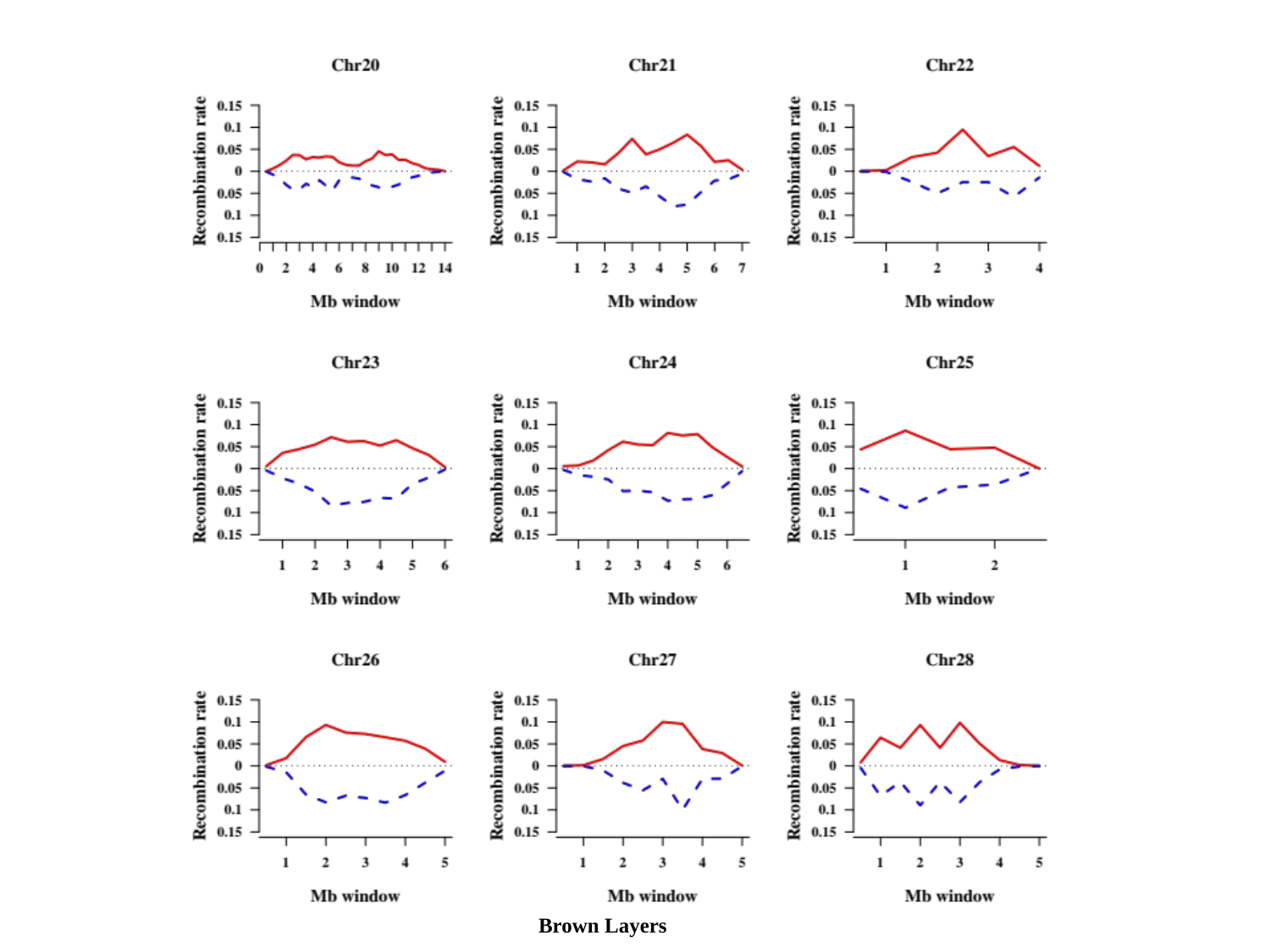

Brown Layers

Supplement: Supplementary file 4 — Figure S4. Variation in recombination rate in males (red solid line) and females (blue dashed line) within 0.5-Mb windows across the 28 autosomes in BL (except GGA16). (PPTX 164 kb) [file 40104_2019_332_MOESM4_ESM.pptx]

White layers

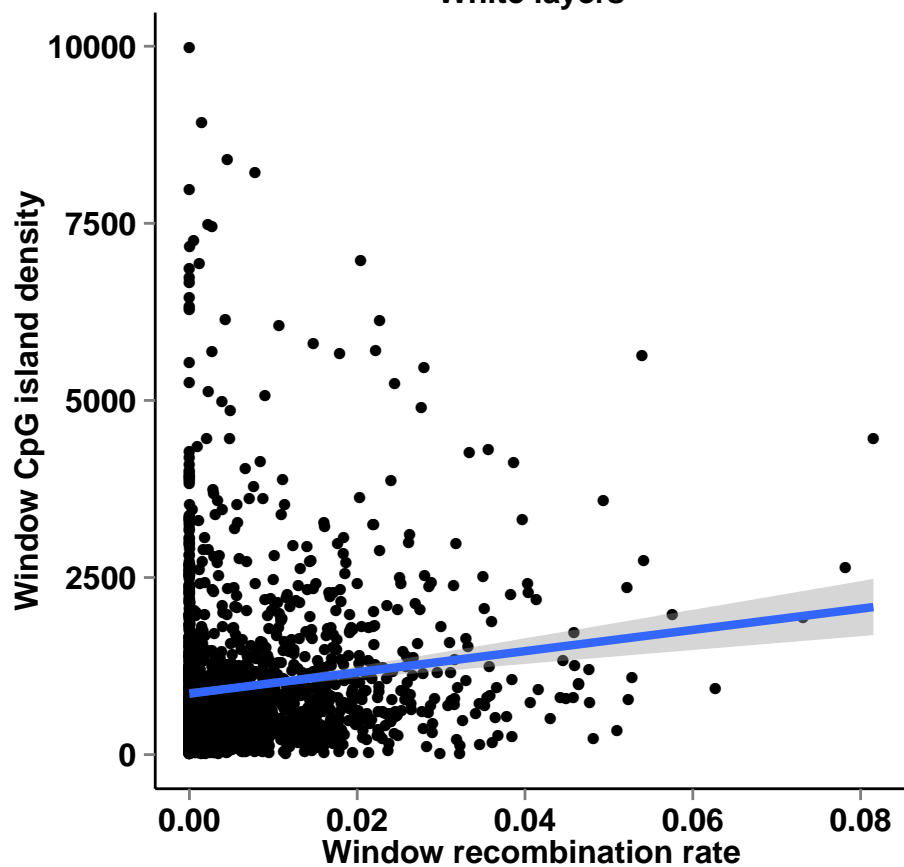

Brown layers

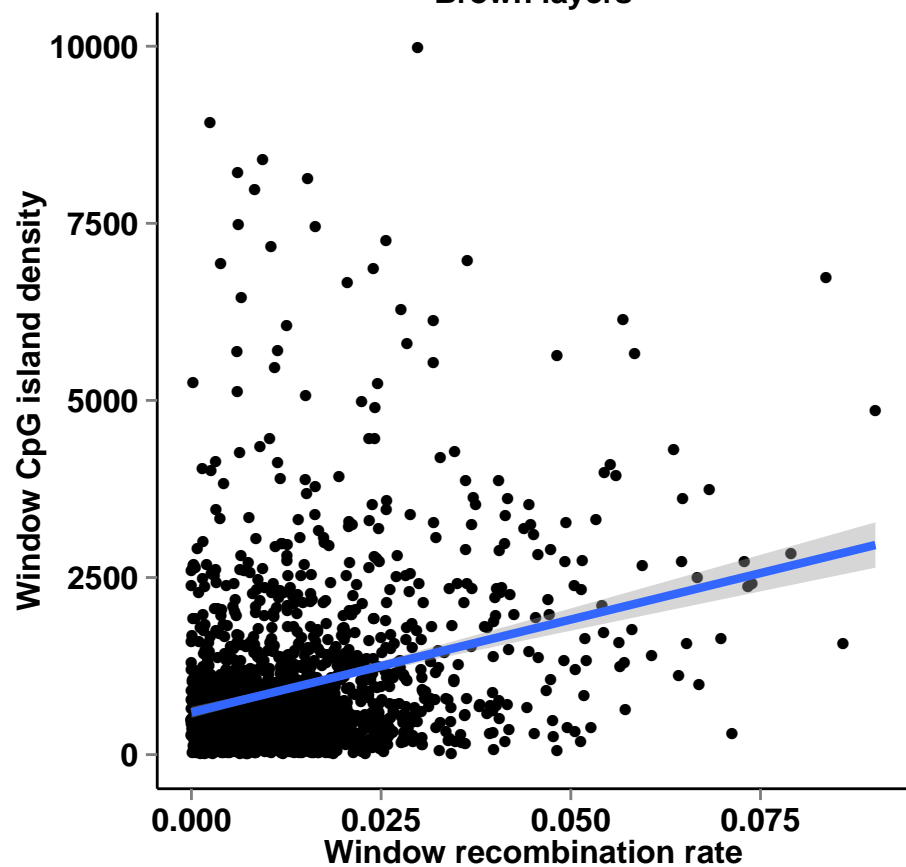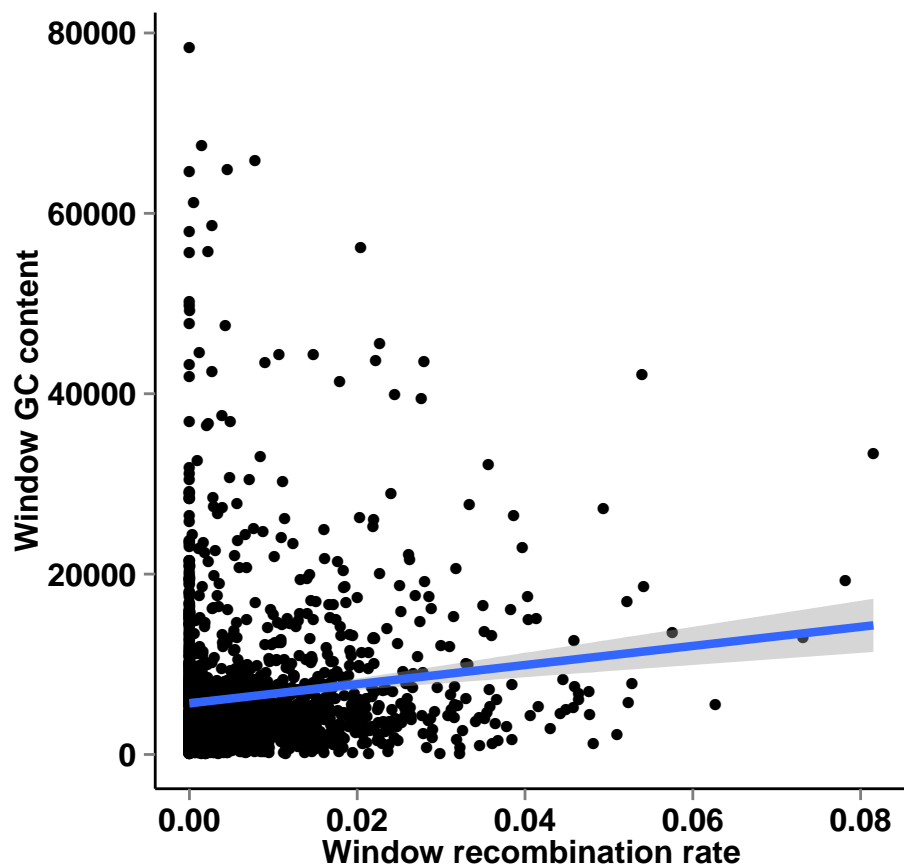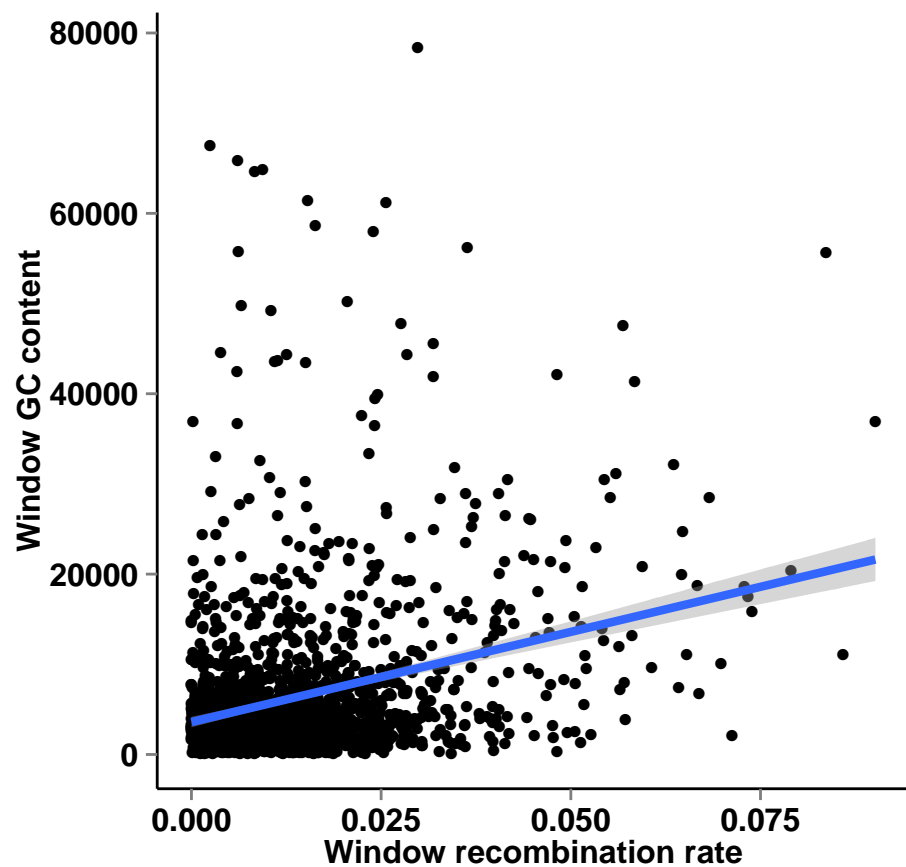

Supplement: Supplementary file 5 — Figure S5. Relationship of GC content and CpG island density with recombination rate within 0.5-Mb window across chromosomes in WL and BL. (PDF 54 kb) [file 40104_2019_332_MOESM5_ESM.pdf]

GRN in white layers

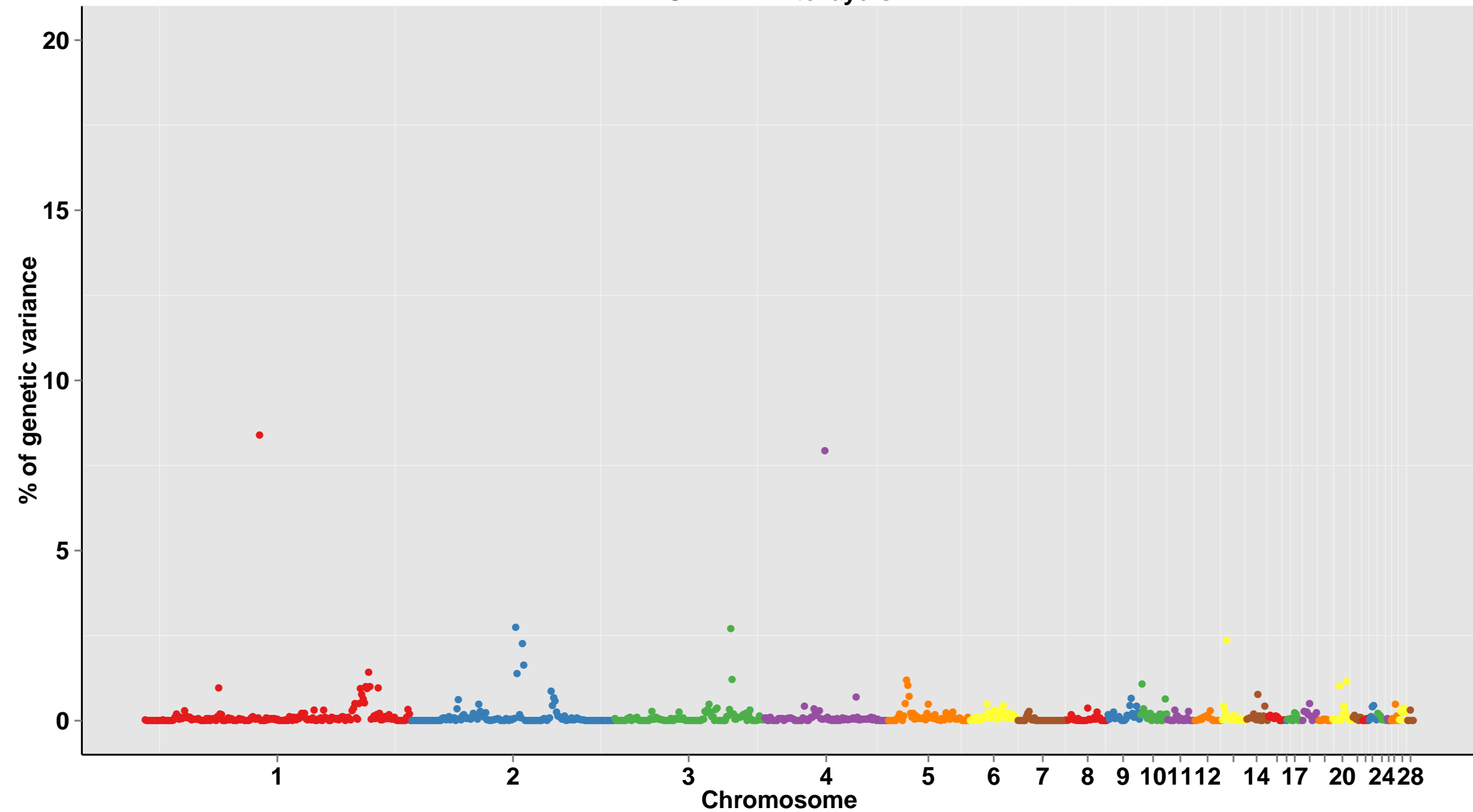

Supplement: Supplementary file 6 — Figure S6. Proportion of genetic variance explained by 1-Mb regions across the genome for GRN in WL. (PDF 10 kb) [file 40104_2019_332_MOESM6_ESM.pdf]

GRN in brown layers

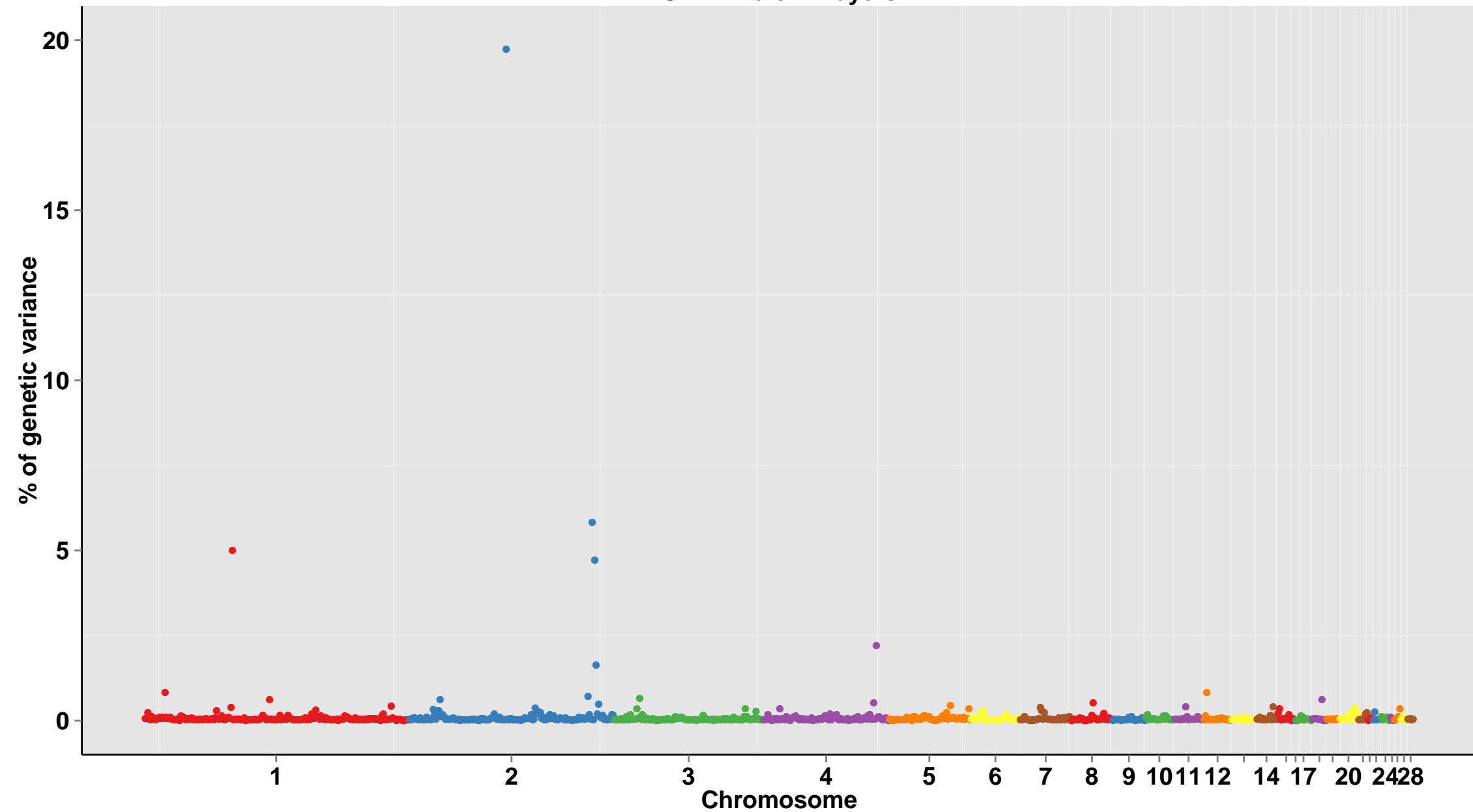

Supplement: Supplementary file 7 — Figure S7. Proportion of genetic variance explained by 1-Mb regions across the genome for GRN in BL. (PDF 10 kb) [file 40104_2019_332_MOESM7_ESM.pdf]
